# Rapid Recovery of Visual Function Associated with Blue Cone Ablation in Zebrafish

**DOI:** 10.1371/journal.pone.0166932

**Published:** 2016-11-28

**Authors:** Gordon F. Hagerman, Nicole C. L. Noel, Sylvia Y. Cao, Michèle G. DuVal, A. Phillip Oel, W. Ted Allison

**Affiliations:** 1 Department of Biological Sciences, University of Alberta, Edmonton Alberta, Canada; 2 Centre for Prions & Protein Folding Disease, University of Alberta, Edmonton Alberta, Canada; 3 Department of Medical Genetics, University of Alberta, Edmonton Alberta, Canada; Wayne State University School of Medicine, UNITED STATES

## Abstract

Hurdles in the treatment of retinal degeneration include managing the functional rewiring of surviving photoreceptors and integration of any newly added cells into the remaining second-order retinal neurons. Zebrafish are the premier genetic model for such questions, and we present two new transgenic lines allowing us to contrast vision loss and recovery following conditional ablation of specific cone types: UV or blue cones. The ablation of each cone type proved to be thorough (killing 80% of cells in each intended cone class), specific, and cell-autonomous. We assessed the loss and recovery of vision in larvae via the optomotor behavioural response (OMR). This visually mediated behaviour decreased to about 5% or 20% of control levels following ablation of UV or blue cones, respectively (P<0.05). We further assessed ocular photoreception by measuring the effects of UV light on body pigmentation, and observed that photoreceptor deficits and recovery occurred (p<0.01) with a timeline coincident to the OMR results. This corroborated and extended previous conclusions that UV cones are required photoreceptors for modulating body pigmentation, addressing assumptions that were unavoidable in previous experiments. Functional vision recovery following UV cone ablation was robust, as measured by both assays, returning to control levels within four days. In contrast, robust functional recovery following blue cone ablation was unexpectedly rapid, returning to normal levels within 24 hours after ablation. Ablation of cones led to increased proliferation in the retina, though the rapid recovery of vision following blue cone ablation was demonstrated to not be mediated by blue cone regeneration. Thus rapid visual recovery occurs following ablation of some, but not all, cone subtypes, suggesting an opportunity to contrast and dissect the sources and mechanisms of outer retinal recovery during cone photoreceptor death and regeneration.

## Introduction

Photoreceptor degeneration within the retina is an irreparable cause of vision impairment and blindness. Broadly, photoreceptors are specialized neurons that absorb light and convert it into electrochemical signals that are transmitted and interpreted by the brain. There are two classes of photoreceptors that mediate vertebrate vision: rods, which are responsible for dim-light vision, and cones, which are responsible for high-acuity, daytime and colour vision. Generating cone photoreceptor cells and managing the rewiring of the remaining retina (remaining cones and remaining second order cells) are primary goals for imagined therapies, because restoring daytime vision would be most impactful on patient quality of life. Unfortunately, this goal remains unrealized and represents a substantial hurdle in deploying stem cells as treatments for blindness.

Retinal stem cell research has focused largely on replacing rod photoreceptors, and impressive progress has been made to transplant rod progenitors, in-so-much that they are able to both survive and to partially restore visual function [1–6]. However, progress has been slow in developing stem cell therapies to replenish lost cone photoreceptors [5]; this may be partially due to the characteristics of common model systems, such as mice, which have rod-dominated retinas suited to their nocturnal habit.

A second major hurdle is that any surviving and/or regenerated photoreceptors must be properly integrated and re-wired into the remaining retinal network. Generated or transplanted photoreceptor cells must integrate into the pre-existing neural retina in order to become functional, and while the success rate of stem cell integration has improved for rod photoreceptors [2], the mechanisms and efficacy are still ambiguous. Arguably, there is a lack of good experimental systems within which researchers can ask questions about retinal plasticity and functional rewiring of regenerating cone photoreceptor cells. We sought to address this issue of functional integration accompanying cone regeneration by engineering novel zebrafish models of cone ablation and using them to measure regeneration of visually mediated behaviours.

Zebrafish are the premier genetic model of cone degeneration and regeneration. Zebrafish retinas are rich in cone photoreceptors of diverse types mediating colour vision (akin to the human fovea), and they have robust regenerative capacity borne upon endogenous retinal stem cells. Teleost fish have the innate capacity to regenerate photoreceptors after loss [7–9]. Teleost fish have contributed much towards the first hurdle described above (regenerating cones, rather than regenerating rods), revealing gene regulatory networks that promote generation of cone photoreceptors; this appears to include interactions amongst *tbx2b*, *gdf6a*, *six7* and thyroid signaling [10–12]. Experiments in mice have produced a more fulsome understanding of this network, revealing Nrl, Nr2e3 and thyroid receptor β as being critical to cone specification [13, 14]. Our comparative approach assessing zebrafish *vs*. mouse photoreceptor development is providing further insights into each system [15], with zebrafish seemingly well-positioned to reveal factors required for generating cone photoreceptors. Separately, we and others have engineered zebrafish to allow conditional ablation of single cone subtypes [16], or of rods [17], and have used these to assess spatial aspects of cone regeneration at the cellular scale. Ablation of UV cones led to a biasing of regeneration to produce significantly more UV cones, restoring the normal neighbor relationships of photoreceptors in the cone mosaic [16]. This was optimistically interpreted as suggesting that a cone-rich environment, such as that present in the zebrafish eye or human fovea, may be best able to support regeneration of cone photoreceptors [16]. Thus frustrated attempts to regenerate daytime vision in rodent models may be solvent: mice might lack a suitable environment for cone generation, and complementary models of cone regeneration that possess high cone densities might be poised to reveal factors required to regenerate cones. Overall, then, zebrafish can contribute to imagining how to best optimize cell-based therapies to regenerate cones and restore daytime vision. However, few examples exist of using fish in regenerative paradigms to address the second hurdle delineated above, i.e. assessing functional integration of regenerated cones while managing the rewiring of any remaining cones (though see [10, 18, 19]).

**Fig 1:**
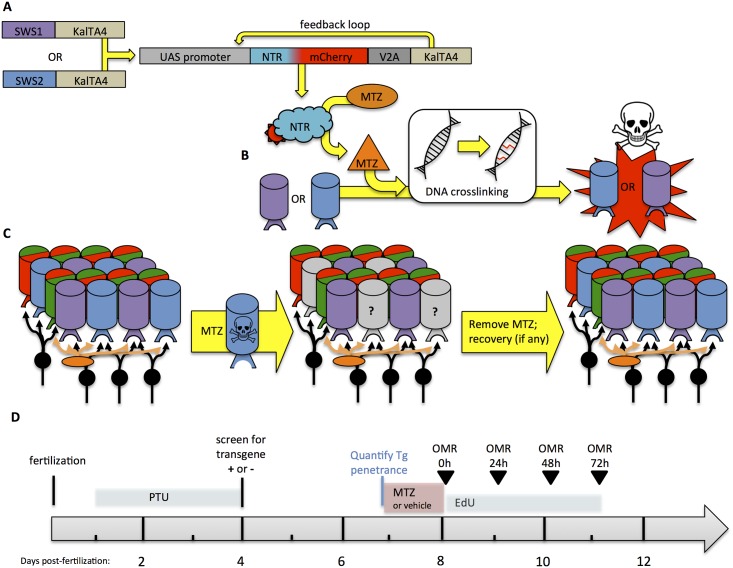
Novel transgenic models permit conditional and targeted cone photoreceptor ablation. Pairs of transgenic zebrafish were engineered so that when they are bred together their progeny express nitroreductase in either UV or Blue cone photoreceptors, allowing for conditional photoreceptor-specific ablation upon addition of a prodrug. **A.** UAS/KalTA4 system of cell specific ablation in either UV or blue cones begins with KalTA4 (an optimized variant of Gal4) being driven by *sws1* or *sws2* promoters, respectively, in different lines of transgenic zebrafish. When these are bred to an additional line of transgenic zebrafish engineered herein, the KalTA4 binds the transgenic UAS promoter to induce expression of the bacterial enzyme nitroreductase (NTR) fused to the fluorescent reporter mCherry. This latter product also encodes KalTA4 protein that breaks away from the NTR-mCherry fusion protein due to inclusion of a 2A peptide; this excess KalTA4 creates a feed-forward loop (“Kaloop”) that maintains its own expression, improving longevity and penetrance of the transgene expression. **B.** Nitroreductase (NTR) converts the prodrug metronidazole (MTZ, applied as a bath treatment in the tank water) into a cell autonomous DNA crosslinking agent, initiating apoptosis in the cone photoreceptors of interest. Thus ablation occurs conditionally, only in the cells expressing the transgene and only in the presence of the prodrug MTZ. **C. Removal of the prodrug MTZ stops the cone ablation and allows retinal recovery to begin.** Left: A small region of the normal adult zebrafish retina is represented, including schematic of the consistent reiterated pattern of cone photoreceptors (cylinders, colours of which represent the four cone spectral subtypes underpinning colour vision) thus forming a predictable mosaic, with second order neurons beneath; Middle: Prodrug MTZ leads to ablation of the specific cone photoreceptor subtype (in this example ablation of blue cones is schematized); Right: Ablation ceases upon removal of the prodrug from the tank water, which we’ve previously shown to induce regeneration of the injured retina from innate retinal stem cells. To be therapeutically useful, the regenerated cones must rewire to the remaining retinal network, including not only other cones but also horizontal cells and bipolar cells (orange & black, respectively). **D. Timeline of experiment.** Larvae were assessed in OMR (optomotor response), assessed for changes in pigmentation in response to UV light, and sampled for histology immediately following application of the prodrug MTZ (at 8 days post-fertilization) and on each day after. 1-phenol-2-thiourea (PTU) was applied to transiently block synthesis of melanin pigment and allow identification of transgenic vs. sibling (wild type) larvae.

Zebrafish have excellent colour discrimination ability [20] driven by four cone photoreceptor types: blue-sensitive, green-sensitive, red-sensitive, and ultraviolet (UV)-sensitive cones [21]. These outputs are compared, fostering tetrachromatic vision [20]. The photoreceptors within the zebrafish retina are organized into a predictable row mosaic [16, 22–24] (Fig 1C). This precisely organized mosaic is a useful tool for the study of retinal physiology and development, as disruptions in the mosaic can sensitively report any patterning errors that occur during development or disease, including via predictable relative abundances of cone photoreceptor types [11, 22, 25].

In order to study factors involved in retinal regeneration and functional recovery of vision, the retina must first be damaged. Photoreceptor damage can be inflicted via numerous means, including surgical removal, chemical lesions, and light ablation [26–29]; however, these methods cause generalized destruction and do not easily target a specific cell type. We sought to engineer a system of conditional ablation using the nitroreductase/metronidazole system to ablate specific cone photoreceptors (Fig 1; see [16]). Nitroreductase converts the prodrug metronidazole into a DNA cross-linking agent thus inducing apoptosis in a cell-autonomous manner [30, 31]. We sought to improve the penetrance and longevity of transgene expression, compared to our recent efforts [16], by implementing the Kaloop feed-forward molecular mechanism [32] in a novel fashion (Fig 1, described below). Moreover, we expressed the construct in an additional cone subtype, enabling a comparison of cellular, molecular and behavioural events following ablation of UV *versus* blue cone photoreceptor cells.

We first characterized the transgenic lines produced with this modified genetic mechanism for efficacy and specificity of ablation. We deployed these fish to investigate the quality and kinetics of functional recovery by assessing the post-ablation recovery of a visually-mediated behaviour. We empirically determined a set of visual stimuli for the optomotor response (OMR) that maximized our ability to document the loss of visual function following ablation of short-wavelength-sensitive cone photoreceptors. Impressively, recovery of visually-mediated behaviour following UV cone ablation consistently occurred over the course of several days, thereby mirroring the timeframe of UV cone reappearance into the retina. Strikingly, recovery of visual function following blue cone ablation occurred quickly, within 24 hours, and was determined to not be mediated by blue cone differentiation or regeneration. This rapid recovery of visual function so soon after loss of blue cones suggests potential for retinal plasticity in the zebrafish retina following ablation of some, but not all, cone photoreceptor subtypes.

## Methods

### Animal ethics statement

Approval for this study, including zebrafish maintenance and breeding, approved as protocol AUP00000077 by the Animal Care and Use Committee: BioSciences, which is an Institutional Animal Care and Use Committee at the University of Alberta, and which operates under the auspices of the Canadian Council on Animal Care.

### Zebrafish care

Adult zebrafish were maintained according to standard procedures [9]. Adult fish were kept in brackish water (1250±50 μS) with 14L:10D lighting conditions provided by standard fluorescent lights, and fed twice daily with either brine shrimp or trout chow. After collection, embryos were transferred into E3 embryo media at 28.5°C and select larvae were treated with 1-phenol-2-thiourea (PTU) 6–8 hours after fertilization until 4 days post-fertilization (dpf) to transiently block synthesis of melanin pigment.

### Engineering transgenic zebrafish to enable conditional cone ablation

Engineering of the novel transgenic fish used herein is described elsewhere [15]. Briefly then, multisite Gateway Cloning and Tol2 systems were used to recombine amongst plasmids and generate transgenic zebrafish by standard methods [33]. We generated a Destination vector, pDestTol2CR2, that identified individual transgenic fish via visualization of their fluorescent red hearts, thereby complementing our transgenic driver lines with green hearts. A KalTA4-loop (“Kaloop”) system, inspired by Distel *et al*. [32], was deployed to enhance expression of our nitroreductase-mCherry fusion protein and recombined from existing plasmids into pDestTol2CR2 [34]. The resulting plasmid, pDestTol2CR2.4UAS:kozak-nfsB-mCherry^DAV-T2A.KalTA4.pA, was designed to express both our previously successful nitroreductase-mCherry fusion protein and the KalTA4 protein as separate peptides. This additional production of KalTA4 then binds the UAS promoter sequence in a feed-forward loop intended both to enhance and to maintain expression of the construct, thereby overcoming gene silencing difficulties observed with the Gal4-UAS system in zebrafish (Fig 1). Expression is initiated only when KalTA4 or Gal4 is produced from an independent transgene (Fig 1). Separately, then, constructs designed to drive expression of KalTA4 (an optimized variant of Gal4) in UV cones or blue cones were generated using promoters of the *sws1* or *sws2* opsins (*opn1sw1* or *opn1sw2*), respectively. These opsin promoters have been described previously and are observed to promote robust and specific expression of various transgenes in UV or blue cones, respectively [16, 35–39].

Transgenic lines stably carrying each of these constructs were identified (Table 1) by screening progeny of injected fish for robust expression of mCherry in either UV or blue cones; this was complemented by screening adult fish for continued robust expression using our custom fluorescent fundoscopy [40]. Two combinations of alleles were used to ablate blue cones, and these were crossed together in experiments described below assessing efficacy and specificity of blue cone ablation.

**Table 1:** Transgenic fish engineered to ablate specific cone subtypes.

Combination of transgenes	Purpose
Tg(sws1:KalTA4)ua3139 Tg(4X-uas:nfsb-mCherry-2A-KalTA4)ua3137	Ablate UV cone photoreceptors
Tg(sws2:KalTA4)ua3136 Tg(4X-UAS:nfsb-mCherry-2A-KalTA4)ua3135	Ablate Blue cone photoreceptors
Tg(sws2:KalTA4)ua3138 Tg(4X-UAS:nfsb-mCherry-2A-KalTA4)ua3137	Ablate Blue cone photoreceptors

### Conditional ablation of photoreceptors

Our conditional cell ablation system expresses nitroreductase (NTR, encoded by *nfsb*) in UV or blue cones (Fig 1). NTR is a bacterial enzyme that converts the prodrug metronidazole (MTZ) into a cell-autonomous DNA cross-linking agent, resulting in cell specific ablation. Ablation of the cone photoreceptors ceases after the prodrug is removed, enabling analysis of retinal recovery. Zebrafish larvae were removed from E3 media at 7 dpf (see experimental timeline Fig 1D), and placed into prodrug metronidazole (10mM; Sigma-Aldrich, St.Louis, MO; Cat. No. M3761 with 0.2% DMSO in E3 media). This experimental group was compared to a control group of non-transgenic sibling larvae receiving this same treatment, whereas a vehicle control group was composed of transgenic fish that received 0.2% DMSO in E3 media. This MTZ treatment was optimized in our previous work [16]. These treatments were ceased after 24 hours (or 48 hours, where indicated), via rinsing larvae three times with E3 media and subsequently rearing in standard conditions with E3 media.

### Quantification of photoreceptor ablation and cell death assay

Retinal cryosections were prepared as previously described [41]. Larvae were fixed overnight at 4°C in 4% PFA, then cryopreserved step-wise in 0.1MPO_4_ solutions with increasing concentrations of sucrose, and embedded in Tissue-Tek O.C.T embedding compound (VWR, Cat. No. 25608–930). Larvae were cryosectioned in 10 μm slices and thaw mounted onto SuperFrost Plus glass slides (Fisher, Pittsburgh, PA; Cat. No. 12-550-15). Tissue was air dried for 30 minutes prior to storage at -80°C.

To determine the effectiveness of specific conditional ablation, transgenic fish were treated with either MTZ or vehicle (0.2% DMSO in E3 media) as described above, then cryosectioned and visualized for mCherry fluorescence. The number of mCherry positive cells was quantified per the length of the outer plexiform layer, to normalize for differences in eye or cryosection size.

The TUNEL assay was used to assess the amount of cell death after conditional ablation in zebrafish larvae (Roche *In situ* Cell Death Detection kit, POD, Cat. No. 11684817910). The TUNEL assay was conducted on cryosections as described previously [16]. Sections were stained with TO-PRO-3 (Invitrogen, Cat. No. T3605; 1/5000 dilution, 30 minute incubation) once the TUNEL reaction ceased. TUNEL-positive cells within the outer nuclear layer of the retina were counted on larval cross-sections and co-localization with mCherry was noted. Cells within the photoreceptor layer that were TUNEL-positive and mCherry-negative were considered to be apoptotic cells that were not blue cones—i.e. other cell types that were undergoing cell death; some such cell death is observed during normal retinal development in zebrafish [42]. Conversely, we quantified TUNEL-positive mCherry-positive cells as being transgenic blue or UV cones, depending on the transgenic model, dying as a result of the prodrug treatment.

### Immunohistochemistry and *in situ* hybridization to characterize photoreceptors before and after ablation

In order to characterize the remaining cones following ablation, immunohistochemistry was performed on 9 dpf larvae that were fixed following 24 hours of MTZ treatment and 24 hours of recovery. Primary antibodies used were 10C9.1 (labels UV cones [16]) and zpr-1 (labels red/green double cones; ZIRC No. ZDB-ATB-081002-43). Secondary antibodies used were AlexaFluor anti-mouse-488 (Invitrogen, Cat. No. A21202) and AlexaFluor anti-rat-647 (Invitrogen, Cat. No. A21472). Antibodies were prepared in 2% normal goat serum in PBS/0.1% Tween-20 (PBSTw) + 1% DMSO. Primary antibodies were diluted to a concentration of 1/100 and secondary antibodies were diluted to 1/1000. Larvae underwent a 5 minute wash in dH2O, followed by a 7 minute wash in -20°C acetone, and a rinse in PBSTw + 1% DMSO. The PBSTw + DMSO was removed from the larvae and blocking solution (10% normal goat serum in PBSTw + 1% DMSO) added for 90 minutes. The larvae were incubated in primary antibodies at 4°C overnight. The larvae were rinsed twice with PBSTw, and secondary antibody was added for overnight incubation at 4°C in darkness. After the secondary antibody incubation, larvae were rinsed with PBSTw then equilibrated in 50% glycerol.

To quantify the photoreceptors remaining after ablation, the left retinas of individual larvae were dissected out and whole-mounted for imaging. ZEN 2010 software (version 6.0, Carl Zeiss AG, Oberkochen) was used with a Zeiss LSM 700 confocal microscope mounted on a Zeiss Axio Observer.Z1 in order to obtain images. To obtain a ratio of remaining, non-ablated photoreceptor subtypes, a 50x50 μm box was drawn onto the images of the whole-mounted retinas using ImageJ (Wayne Rasband, National Institutes of Health, Bethesda MD; http://rsbweb.nib.gov/ij/index.html). UV and double cone photoreceptors were counted within this box.

To quantify the number of UV cones expressing the nfsb-mCherry transgene prior to ablation, 7dpf larvae were fixed and processed for immunohistochemistry with the anti-UV-opsin antibody 10C9.1, as described above. In order to quantify the number of blue cones expressing the nfsb-mCherry transgene prior to ablation, 7dpf larvae were fixed and processed for fluorescent double-label *in situ* hybridization as per previous protocols [22] using *sws2* opsin and mCherry antisense riboprobes. Antisense riboprobe vs. full length *sws2* opsin were labelled with DIG and produced as per published protocols [22], and antisense riboprobe vs. mCherry were labeled with FLR and raised against 500 bp of mCherry cDNA sequence as templated from a pCS2+ vector cut with HindIII. Imaging again used a Zeiss LSM 700 confocal microscope mounted on a Zeiss Axio Observer.Z1. All cones labelled for the particular opsin subclass, all cells containing mCherry, and cells containing both labels were quantified across entire retinas using ImageJ. No differences in the consistency of co-labelling were obvious across the different regions of the retina.

### Quantifying retina proliferation

Following ablation of blue or UV cones as above, EdU (5-ethynyl-2´-deoxyuridine) was added to some larvae to assess if cone ablation had any effect upon proliferation. After removal of MTZ, larvae were treated with 100μM EdU in E3 media for 96 hours, from 8 dpf through 12 dpf (see Fig 1) with daily changes replacing with fresh E3 media containing EdU. At 12 dpf, the fish were washed three times out of EdU media, returned to E3 media and sacrificed at 14 dpf. Fixed larvae were cryosectioned as above, and EdU+ cells were visualized with a fluorescent signal as per manufacturer’s protocol (Baseclick GmbH, Munich Germany; BCK488-IV-IM-S). EdU+ cells were counted within the outer or inner nuclear layers (ONL or INL) or the ciliary marginal zone (CMZ), and these counts were normalized against the length of the retinal section by dividing the counts by the length of the outer plexiform layer.

### Optomotor response assay

The optomotor response (OMR) is a visually mediated behaviour observed in larval zebrafish older than 6 dpf [43]. For the OMR assay, the apparatus consisted of a computer monitor (IBM 6734-HBO ThinkVision) laid horizontally that delivered visual stimuli (animated moving bars), with 10 troughs (custom; 30cm x 1.4cm x 1.0cm, composed of 1.7mm thick transparent acrylic sheets chemically bonded together) sitting on top of the monitor, arrayed with their long axes orthogonal to the moving stripes. Each trough was filled with 30°C system water and contained a single zebrafish larva. The location of the larval zebrafish was tracked using a digital camera (Cyber-shot DSC-HX50V, Sony, Tokyo) mounted overhead of the computer monitor. The moving bar pattern parameters were constant (velocity: 1.33cm/s, width of the bars 1.35cm, and frequency 0.49Hz), generated in PowerPoint (Microsoft, Redmond, Version 14.5.2) on a MacBook Pro connected to the monitor via VGA cable. Each stimulus was characterized via spectrometer (USB4000-UV-VIS: Ocean Optics, Dunedin) through an optical fiber with a cosine-corrector (CC-3-UV-S: Ocean Optics Inc., Dunedin FL) held at the typical position of the fish (through an acrylic sheet equivalent to the trough containing the larvae). The spectrometer was calibrated using a radiometric calibration source (HL-2000-CAL, Ocean Optics Inc.). Data regarding stimuli parameters was assembled using *SpectraSuite* software (Ocean Optics Inc.) and *Microsoft Excel* (Microsoft, Redmond WA).

Prior to experimental recording, larval zebrafish were acclimated to an OMR stimulus in the reverse direction for 1 minute. The duration of the experimental forward stimulus was 1 minute, after which the position of the larvae was recorded on a white background. Larvae were exposed to a series of optomotor stimuli in random order with two technical replicates per larva, which were averaged as one biological replicate. Larval zebrafish were selected randomly from a treatment group population. Only fish that responded to pipette suction by exhibiting attempted escape movements were used in the OMR assay. Behavioural recordings were taken from 8–11 dpf fish (see timeline in Fig 1D). Behavioural analysis did not proceed past 11 dpf because the health of larval zebrafish at this age began to be variable. The position of each larvae relative to its initial location was quantified using ImageJ. In some histograms, average distances moved were normalized to the average value from control larvae (MTZ treated wild type non-transgenic siblings) at each age.

Validation of the OMR assay first relied on assessing if zebrafish moved more to the stimuli described above relative to the same stimulus played in reverse, wherein the latter stripes moved towards the start zone of the arena (described in S1 Fig). We predicted that if larvae were responding to the moving stimulus they would move a greater distance through the arena with forward moving stripes ‘motivating’ swimming compared to larvae that were following stripes in reverse which would remain at the start of the arena. A further validation of our OMR by comparing responses to alternative stimuli was performed by recording responses to a high contrast stimulus of black & white bars.

Second, we assessed if our transgenic zebrafish treated with MTZ exhibited deficits in movement. Larval transgenic *Tg[sws2*:*nfsb-mCherry]* zebrafish at 9dpf were treated with prodrug MTZ or vehicle DMSO as above (= 0h after MTZ). Observer was blinded to treatment groups until after statistical comparison of outcomes was performed. Larvae were arrayed in troughs on a horizontally-placed computer monitor exactly as described in the OMR assay, except that a white screen was displayed. A tapping stimulus was applied to the computer monitor using a rotating platform (VWR #SO500) that tapped the monitor at a rate of 1 Hz. Positions of larvae were noted relative to a ruler after 5 minutes of stimulus application. Initial experiments suggested that larvae moved towards such tapping stimuli, so the tapping was applied at the end of the trough furthest from the larval start position.

A further test of potential movement deficits used fish treated exactly as per those above in the Touch Evoked Escape Response. Larval transgenic *Tg[sws1*:*nfsb-mCherry]* zebrafish at 9dpf treated with prodrug MTZ or vehicle DMSO were placed in the approximate centre of a 100 mm petri dish on a horizontally-placed monitor of the same model above. Larvae were lightly touched on their tail with fishing line to induce an escape response. Larvae were touched three times or until they moved more than one centimeter, whichever occurred first. Motion of larvae was recorded from above with larvae on the same computer monitor used for OMR (IBM 6734-HBO ThinkVision) similarly oriented (laid horizontally) and projecting a white screen. A video camera (Color CCTV Camera WV-CL930, Panasonic Tokyo) was mounted overhead connected to a PC running EthoVision^®^ XT7 software (Noldus, Wageningen, Netherlands), via a video capture card (Picolo H.264; Euresys, San Juan Capistrano, CA), as described previously [44]. Ethovision software was used to quantify the distance larvae travelled during the touch evoked escape response. Observer was blinded to treatment groups until after statistical comparison of outcomes was performed.

An additional test of larval movement used the previously established Spontaneous Swim Assay [45]. Larval transgenic zebrafish *Tg[sws1*:*nfsb-mCherry]*, *Tg[sws2*:*nfsb-mCherry]* were tested at 8dpf following 24 hour prodrug treatment as previously described. The behavioral apparatus was enclosed to minimize luminance motion cues. Arena configuration and imaging via Ethovision software was as described immediately above. The behavioral arena was a petri dish (Fisher Scientific 100mm x 15 mm) filled with 30ml of brackish water (1250±50 μS) heated to 30°C. Each petri dish contained 10 larval zebrafish at a time. Zebrafish were selected randomly from a treatment population, and presence of a robust escape response upon attempt to capture each larva was required. Zebrafish acclimated for 20 minutes prior to behavioral recording. Behavioral recordings occurred over 10 minutes; video recording was generated using *Ethovision* (Noldus Information Technologies Inc., Leesburg, VA, version XT 10.0). Frames were extracted from the video at a rate of 1 per second using *VLC Media Player* (VideoLan Organization, Paris, Version 2.2.2). The video frames were analyzed using ImageJ stack sorter plugin in conjunction with a stack pixel subtraction plugin (Delta F down, Tony Collins, McMaster Biophotonics Facility, MBF-plugin collection). The subtraction plugin filtered images to identify pixels that changed in value relative to the previous frame; thereby only larvae that moved are included (i.e. stationary fish are subtracted). A Z-projection of the subtracted video frames created a movement profile of the entire 10 minute recording for display (S3 Fig). The number of fish present on each frame was quantified as a movement event using particle analysis in ImageJ, and the rate of movement over 10 minutes was then tabulated using Microsoft Excel. The movement events for ten fish were thus averaged to create a single statistical unit (n = 1), and multiple such units were collected for each treatment.

Third, we validated our OMR stimulus by assessing if it could detect visual deficits in larval zebrafish treated equivalently to our cone ablation models but instead harbouring known deficits in vision. We performed OMR on *gdf6a*^*s237*^ mutant microphthalmic larvae and their siblings. These mutants have a reduced abundance of blue cones relative to other cone types [11], photoreceptor degeneration [46], small eyes, increased body pigmentation via melanin dispersion [46], and reduced response to conventional black and white OMR stimuli [46]. These mutants also exhibit progressive neuromuscular deficits [47], but this only appears in adult fish.

A second group of larvae with known visual deficits was used to validate our OMR: wild type larvae were raised equivalently to our cone ablation models but were instead treated with intense UV light to acutely photobleach and subsequently ablate photoreceptors. An industrial array of 405 nm diodes (#1167593 and controller #1359255, Locite, Rocky Hill CT) was inverted to deliver intense (600 mW/cm^2^) UV light in a skyward direction. Larval zebrafish (9 dpf) in E3 media and in a 35 mm Pyrex petri dish were placed directly onto the diode array (LED array dimensions 100 X 152 mm). UV light was delivered to the larvae for 30.0 seconds. We presumed that this intense light, delivered from the same direction as the OMR stimulus, would bleach the photoreceptors and thus represent a visual deficit. Larvae treated with UV light were compared to untreated siblings in the OMR assay with red and blue bars.

An additional test of whether our OMR can detect visual deficits examined larvae with cones ablated by toxic doses of light, akin to similar approaches in adult zebrafish. The larvae treated with intense UV light described immediately above were grown for an additional day (to 10 dpf) and again compared to untreated siblings in the OMR assay with red and blue bars. These larvae were fixed for histology at the end of the OMR, and cryosections confirmed the expected damage of retinal photoreceptors (S2 Fig), which exhibited severely truncated outer segments and pyknotic nuclei.

### Quantifying photoreception via body pigmentation and melanin dispersion

Our assay of melanin dispersion quantifies changes in body pigmentation that occur in response to light. It was used as a measure of ocular photoreception that was not dependent on larval swimming ability, bypassing any potential confounds of our transgenic and pharmacological interventions upon the neuromuscular system or locomotion. Larval zebrafish were positioned in a Pyrex petri dish (100 mm) within 4% methylcellulose made in embryo media, and immobilized using low-gelling-temperature agarose (Sigma, Cat. No. A4016) made in embryo media, covered by a layer of normal (stiffer) agarose (Invitrogen, Cat. No. 6500) made in embryo media, and covered with 15 mL embryo media. Larvae were positioned on a platform 35 cm above an array of 405 nm diodes (Locite, described above), with diodes facing skyward such that the light was directed at the ventral side of the larvae. A hole in the platform restricted the light from reaching the petri dish except in the region containing the larvae. Images were captured by briefly moving the preparation of larvae from this array to be imaged on a dissection microscope (Leica MZ16F with Olympus camera DP72 and DP2-BSw image capture software). Larvae were immobilized in normal room lighting, and then dark adapted for 30 minutes, after which time the first image was collected. Larvae were subsequently exposed to UV light and imaged every 10 minutes for 30 minutes. Individual larvae were not assessed on multiple days. Quantification of pigmentation was performed by binarization of the images (Adobe Photoshop CC 2014) to create a binary representation of each larvae at each time point (described in Results). Quantification of dark pixels in binarized images was performed in ImageJ. A melanin index was attained by arbitrarily setting the pixel value for each individual dark-adapted larva to 1. At subsequent time points the melanin index was calculated for each individual larva, by forming a ratio of the pixel value relative to its dark-adapted state. Larvae that darkened during light exposure via melanin dispersion are represented by a melanin index greater than one.

### Statistical analysis

Photoreceptor counts following ablation were assessed using two-way ANOVA with a Bonferroni post-hoc test. Photoreceptor ratios were assessed using the Kruskal-Wallis Test. Abundance of EdU+ cells was compared by Kruskal-Wallis analysis of variance with Dunn’s post-hoc pairwise comparisons. TUNEL data were assessed via pairwise comparison using the Dwass-Steel-Chritchlow-Fligner Test. OMR responses and pigmentation assays were assessed via One- or two-way ANOVA with post-hoc Tukey test. All tests were run in SYSTAT 13 (Systat Software, San Jose CA) or Prism Software (version 7.0 for Mac, GraphPad Software, La Jolla CA). Data is presented as mean ± 1 standard error (SE) of the mean except where noted.

## Results

### Characterizing the cell specific ablation of blue or ultraviolet sensitive cone photoreceptors

Conditional ablation of UV or Blue cones was induced in transgenic zebrafish through the addition of the prodrug metronidazole (MTZ) to the tank water. Cell specificity of ablation was conferred by restricting expression of the bacterial enzyme nitroreductase (NTR) to specific cone photoreceptor subtypes. In the presence of the bacterial enzyme NTR, the prodrug MTZ is chemically reduced, converting it to a cell-autonomous DNA cross-linking agent and thus initiating apoptosis. When the prodrug is removed from the tank water, cone ablation ceases and regeneration of the ablated subtype, if any, can occur.

**Fig 2:**
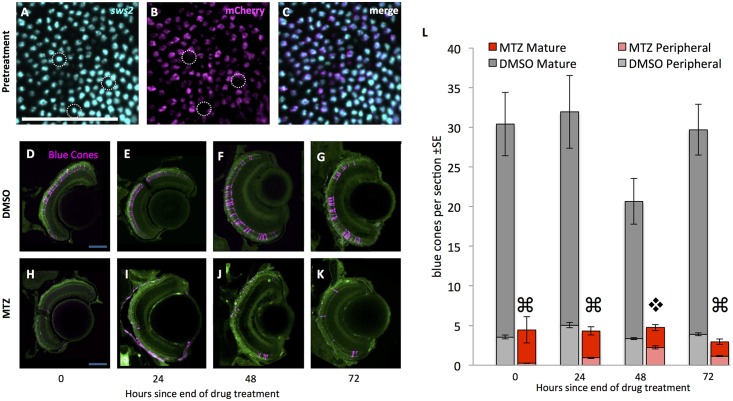
Blue cone photoreceptors are efficiently ablated upon addition of prodrug. (A-C) Prior to treatment with prodrug metronidazole (MTZ) most blue cones (cyan) express the nfsb-mCherry transgene (magenta); some exceptions are noted (dotted lined circles). Wholemount retinae labelled with antisense riboprobes against *sws2* blue opsin and against mCherry viewed *en face* (tangential plane). 78±2% of blue cones express the nfsb-mCherry transgene, considering all 1430±60 blue cones across the entire retina of 7dfp larvae (n = 4), the age at which they begin to receive MTZ in our ablation paradigm. **D-K.** Retinal sections of larval zebrafish expressing the transgenes *Tg[sws2*:*KalTA4; UAS*:*nfsb-mCherry-KalTA4*] such that blue cones contain the nitroreductase-mCherry fusion protein (pseudocoloured magenta). MTZ was applied via bath treatment for 24 hours beginning at 7 days post-fertilization (dpf); the end of this 24 hour period is denoted in the text and figures as zero hours since the end of drug treatment. Top row: Blue cones are intact in retinal sections of transgenic larvae treated with the vehicle control (DMSO, applied at 7 dpf; time 0h = 8dpf). Bottom row: Blue cones are ablated, as shown in retinal sections of transgenic larvae treated with the prodrug. A decrease in the number of mCherry-positive blue cones (magenta) is apparent through 48 hours post ablation, followed by observation of mCherry-fluorescing blue cones at the ciliary marginal zone (panels G & H). Auto-fluorescence is included for spatial reference (green). **L.** Blue cones were reduced in abundance four-fold after prodrug MTZ treatment (red bars) relative to vehicle control (DMSO; grey bars). Abundance of cones in the peripheral retina is represented by lighter grey or red bars. Blue cones were gradually added to the periphery (light red bars) on the days following MTZ. Quantification considered cones in the peripheral retina and mature retina (⌘ indicates p<0.0001 when comparing blue cone abundance in mature retina treated with MTZ relative to DMSO, and p<0.05 when comparing peripheral retina treated with MTZ relative to DMSO; ❖ denotes significant difference (p<0.0001) when comparing blue cones in mature retina with MTZ relative to DMSO, but peripheral blue cone abundance is not significantly different between treatments. n≥5 fish for each treatment). Scale bars are 50 μm.

**Fig 3:**
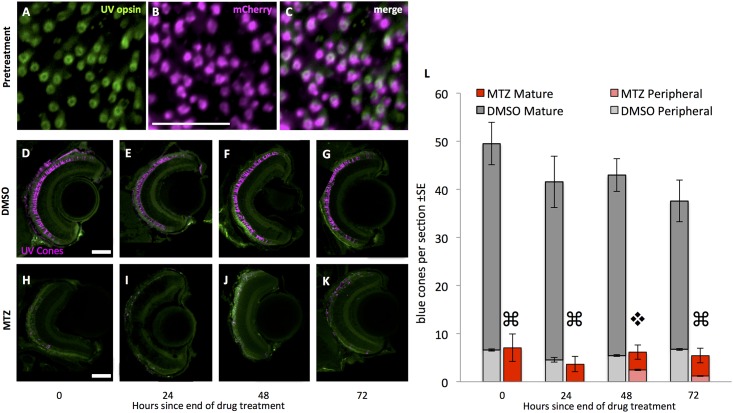
UV cones are efficiently ablated using our model of cell specific ablation. Prior to treatment with prodrug metronidazole (MTZ), most UV cones (green, labelled with antibody 10C9.1 against UV opsin) express the nfsb-mCherry transgene (magenta). **A-C.** Wholemount retinae viewed *en face* (tangential plane). 81±4% of UV cones express the nfsb-mCherry transgene, considering all 1622±96 UV cones across the entire retina of 7 days post-fertilization (dpf) larvae (n = 5), the age at which they would begin to receive MTZ in our ablation paradigm. Scale bar in panel J is 20 μm. **D-K.** Larval zebrafish expressing the transgenes *Tg[sws1*:*KalTA4; UAS*:*nfsb-mCherry-KalTA4*] in the UV cones, such that UV cones contain the nitroreductase-mCherry fusion protein (pseudocoloured magenta). Top row: UV cones are intact in retinal sections of transgenic larvae treated with the vehicle control (DMSO, applied at 7 dpf). Bottom row: UV cones are ablated, as shown in retinal sections of transgenic larvae treated with the prodrug MTZ (applied via bath treatment for 24 hours beginning at 7 dpf; the end of this 24 hours is labeled as zero hours since the end of drug treatment). A decrease in the number of mCherry-positive UV cones (magenta) is apparent through 48 hours post ablation, followed by observation of mCherry-fluorescing blue cones at the ciliary marginal zone (panel H). Auto-fluorescence is included for spatial reference (Green). Scale bars are 50 μm. **L.** UV cones were reduced in abundance four-fold after prodrug MTZ treatment (red bars) relative to vehicle control (DMSO; grey bars). Abundance of cones in the peripheral retina is represented by lighter grey or red bars. UV cones were gradually added to the periphery (light red bars) on the days following MTZ. Quantification considered UV cones in the peripheral retina and mature retina (⌘ indicates p<0.0001 when comparing UV cone abundance in mature retina treated with MTZ relative to DMSO, and p<0.05 when comparing peripheral retina treated with MTZ relative to DMSO; ❖ denotes significant difference (p<0.0001) when comparing UV cones in mature retina with MTZ relative to DMSO, but peripheral UV cone abundance is not significantly different between treatments. n≥5 fish for each treatment).

Expression of each construct and subsequent ablation of the cones was confirmed by observing an mCherry fluorescent reporter, which was fused to NTR, in the cone photoreceptors. Expression of the fusion protein was mediated by KalTA4, the expression of which was driven in specific cells using either a UV cone specific promoter (*sws1*) or blue cone specific promoter (*sws2*) in conjunction with the KalTA4 sensitive UAS promoter (Table 1). A second copy of KalTA4, produced alongside NTR under the UAS promoter, creates a feedback loop, maintaining expression of the construct into adulthood (Fig 1). Transgenic lines denoted Tg(*SWS1*:*KalTA4;UAS*:*nfsb-mCh-2A-KalTA4*) and Tg(*SWS2*:*KalTA4;UAS*:*nfsb-mCh-2A-KalTA4*) (various alleles, see Table 1, abbreviated in the text as “*Tg[sws2*:*nfsb-mCherry]*”) expressing the NTR-mCherry fusion proteins in the appropriate cell types were identified (Figs 2 and 3).

Blue cone ablation in larval zebrafish was quantified following a 24 hour MTZ prodrug exposure beginning at 7 days post-fertilization (dpf) (timeline in Fig 1, results in Fig 2). Prior to ablation, approximately 80% of the blue cones contained the nfsb-mCherry transgene (Fig 2A and 2C). MTZ exposure caused a significant four-fold reduction in the abundance of mCherry-positive cells immediately following the 24-hour exposure to MTZ (Two-Way ANOVA, p<0.001). Following MTZ exposure, the retina contained remnant mCherry-positive bodies that were dysmorphic relative to healthy cone photoreceptors (Fig 2D–2K). Intact mCherry-positive cone photoreceptor cells were first observed in the retina as early as 48 hours following ablation, predominantly in the developing peripheral retina. These early cells can likely be attributed to the persistent growth of the retina from the ciliary marginal zone (CMZ).

UV cone ablation using the optimized iteration of the transgene was also successful. Prior to ablation, approximately 80% of the UV cones contained the nfsb-mCherry transgene (Fig 3A–3C). The abundance of mCherry-positive cells was again significantly reduced immediately following prodrug MTZ exposure (Two-Way ANOVA, p<0.001) (Fig 3).

Ablation of either blue or UV cones led to increased abundance of EdU+ cells in the retina, consistent with increased proliferation of endogenous retinal stem cells. The abundance of EdU+ cells in the INL was 10- or 5-fold higher when ablating blue or UV cones, respectively, relative to sibling transgenic fish receiving DMSO vehicle (S4 Fig; Kruskal-Wallis test, p<0.01 & p<0.05, respectively). The number of EdU+ cells in the ONL was 4- or 3-fold higher when ablating blue or UV cones, respectively, relative to sibling transgenic fish receiving DMSO vehicle (S4 Fig; Kruskal-Wallis test, p<0.001 & p<0.05, respectively). The abundance of EdU+ cells in the CMZ approximately doubled in following ablation of either cone type, though this was significant only for blue cone ablation (S4 Fig; Kruskal-Wallis test, p<0.05). In none of these three populations of proliferating cells (ONL, INL or CMZ) was the abundance of EdU+ cells significantly different between blue vs. UV cone ablation in this small sample (S4 Fig). This increased proliferative activity following cone ablation is a potential source of cone regeneration, though further tests would be required to assess if the proliferating cells differentiate to become photoreceptors.

### Targeted ablation of cones using the nitroreductase method is cell autonomous

**Fig 4:**
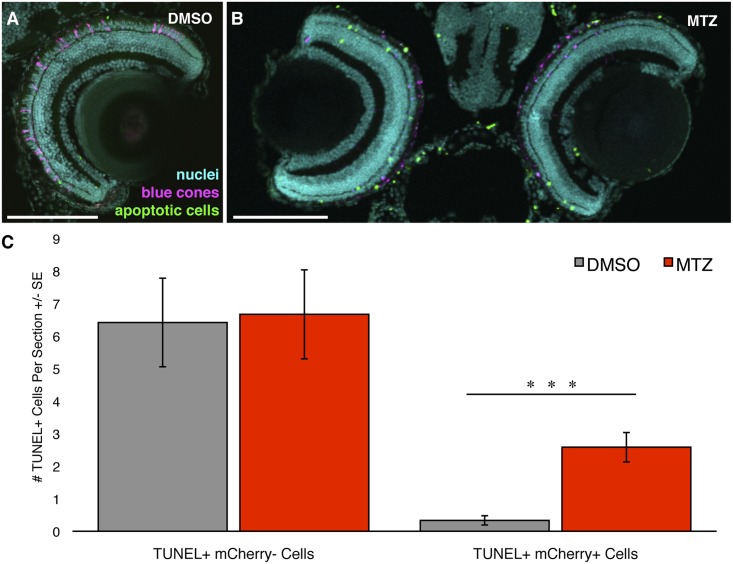
Ablating blue cones does not cause any detectable off-target cell death. Transgenic zebrafish engineered for blue cone ablation were labeled for apoptosis using TUNEL to examine death of target photoreceptors (blue cones) and any effects on adjacent photoreceptors. **(A, B)** Zebrafish larvae that had received either vehicle control (DMSO) or prodrug metronidazole (MTZ) treatment, respectively, sacrificed at 9 days post fertilization (dpf). Blue cones expressing mCherry are pseudocoloured in magenta, TUNEL labelling is represented in green, nuclei are in cyan. Intact blue cones are not detectable in the prodrug treated larvae. **(C)** Quantification of the number of TUNEL-positive cells per section within the ONL. The number of TUNEL+ mCherry- cells did not differ significantly between treatment groups, indicating no detectable off-target cell death. The number of TUNEL+ mCherry+ cells differed significantly between groups, as was expected since the prodrug treatment is designed to induce apoptosis in the mCherry+ blue cones of this transgenic model. Scale bars are 100μm. *** = p<0.001. n = 12 & 11 fish for the DMSO & MTZ treatment groups, respectively.

We next investigated the cell autonomy of the blue cone ablation, and whether it has a toxic bystander effect, by assaying both the death of the targeted photoreceptor cells and any potential increase in the death of nearby cells. TUNEL labeling for cell death was performed on larval cryosections, revealing labelling of dysmorphic cones found only in MTZ treated transgenic larvae (Fig 4B). The abundance of TUNEL-positive mCherry-negative cells was not significantly different between the treatment groups; rather, both groups had a nearly identical amount of non-target apoptotic cell death within the outer nuclear layer (n = 11 and 12 fish; Fig 4C). This non-target cell death may represent the normal cell death that is known to occur in the developing zebrafish retina [42]. In contrast, there was a significant eight-fold increase (p<0.001) in the number of TUNEL-positive mCherry-positive cells in the drug treated group compared to the vehicle control treated group. This observed co-localization between TUNEL labelling and mCherry expression, strictly following MTZ exposure, is evidence that this cone specific ablation model conditionally ablates the target cell (blue cones) and not adjacent cells.

**Fig 5:**
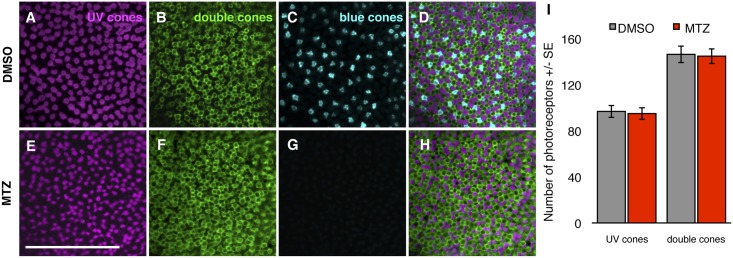
Blue cone ablation does not cause alterations in the abundance of non-target cone subtypes. **(A-H)** Transgenic zebrafish retinae labelled with antibodies against UV and double cones. Top Row: Treated with vehicle control DMSO; Bottom Row: treated with prodrug metronidazole (MTZ) to induce ablation of blue cones. Retinas are visualized as whole mounts, wherein UV cones are labeled with antibody 10C9.1, double cones are labeled with antibody zpr1, and blue cones are filled with mCherry (pseudocoloured to cyan) fused to nitroreductase. Note that in (G) blue cones are absent due to application of prodrug MTZ. **(D, H)** Merged image showing the larval mosaic. Scale bar is 50 μm. **(I)** The numbers of UV and double cone photoreceptors obtained from DMSO and MTZ treated zebrafish larvae are indistinguishable, i.e. ablating blue cones does not detectably disrupt adjacent cones. n = 12 fish for both treatments.

Because TUNEL labeling captures cell death only within a narrow window of time relative to tissue fixation, we also investigated bystander toxicity by assessing the numbers of remaining cone photoreceptors. We reasoned that any cell death among non-target photoreceptors could be detected as a perturbation in the local quantity of double (red and green) cones or UV cones, following blue cone ablation. The quantity of remaining cones of various subtypes was very stable following ablation of blue cones (n = 12 fish per genotype; Fig 5), consistent with the predicted cell-autonomous effect of the nitroreductase ablation system. The abundances of UV and double cones were not different between the vehicle control and MTZ-treated groups (Fig 5I). Comparing the abundance of double cones to UV cones as ratios (# double cones/# UV cones) revealed values that were nearly indistinguishable between treatment groups: 1.52 ± 0.04 and 1.54 ± 0.06 (n = 12 fish per genotype) for DMSO and MTZ treatment groups respectively. The values of these ratios are in good agreement with past results, as it was previously determined that the larval mosaic has more green and red cones than UV cones, though at values lower than the 2:1 ratio of adult fish cone mosaics [22, 25]. This data further supports that MTZ treatment does not result in a toxic bystander effect and that our genetic model of conditional cone ablation is cell autonomous.

**Fig 6:**
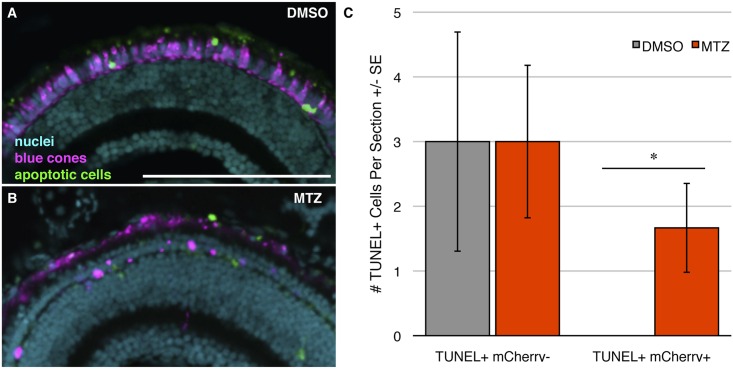
Ablating UV cones does not cause any detectable off-target cell death. Transgenic zebrafish engineered for UV cone ablation were labeled for apoptosis using TUNEL to examine death of target photoreceptors (UV cones) and any effects on adjacent photoreceptors. **(A, B)** Zebrafish larvae that had received either vehicle control (DMSO) or prodrug metronidazole (MTZ) treatment, respectively, sacrificed at 9 days post fertilization (dpf). UV cones expressing mCherry are pseudocoloured in magenta, TUNEL labelling is represented in green, nuclei are in cyan. Intact UV cones are not detectable in the prodrug treated larva. **(C)** Quantification of the number of TUNEL-positive cells per section within the ONL. The number of TUNEL+ mCherry- cells did not differ significantly between treatment groups, indicating no detectable off-target cell death. The number of TUNEL+ mCherry+ cells differed significantly between groups, as was expected since the prodrug treatment is designed to induce apoptosis in the mCherry+ UV cones of this transgenic model. * = p<0.05. n = 5 & 9 fish for the DMSO & MTZ treatment groups, respectively. Scale bar is 100μm.

**Fig 7:**
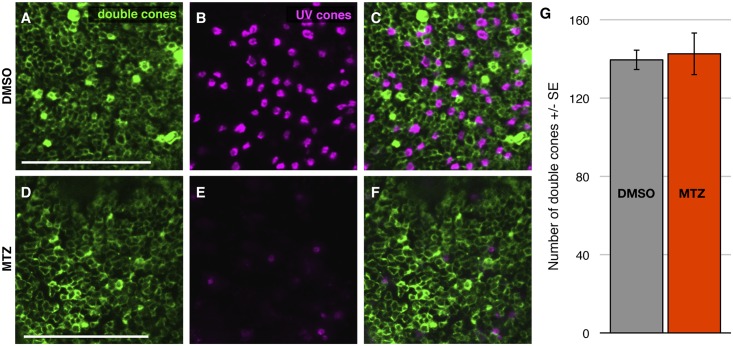
UV cone ablation does not cause alterations in the abundance of non-target cone subtypes. **(A-F)** Transgenic zebrafish retina labelled with antibodies against double cones. Top Row: Treated with vehicle control DMSO; Bottom Row: treated with prodrug metronidazole (MTZ) to induce ablation of UV cones. Retinas are visualized as whole mounts, wherein double cones are labeled with antibody zpr1, and UV cones are filled with mCherry (pseudocoloured to cyan) fused to nitroreductase. Note that in (E) UV cones are greatly reduced in abundance due to application of prodrug MTZ. **(C, F)** Merged image showing the larval mosaic. Scale bar is 50 μm. **(I)** The numbers of UV and double cone photoreceptors obtained from DMSO and MTZ treated zebrafish larvae are indistinguishable, i.e. ablating blue cones does not detectably disrupt adjacent cones. n = 6 or 5 fish for DMSO and MTZ treatments, respectively.

Our new model of UV cone ablation, ua3137;ua3139 *Tg(SWS1*:*KalTA4;UAS*:*nfsb-mCh-2A-KalTA4*) (Table 1), is an improved iteration of our previously engineered and characterized line ua3016;c264 *Tg*(*SWS1*:*Gal4-VP16;UAS-E1b*:*nfsb-mCherry*) [16], in-so-much that it has increased penetrance and longevity of the transgene expression; we interpret this to mean that the Kaloop strategy was successful in improving the ability to label and ablate cones in older zebrafish larvae. The previously characterized line had no toxic bystander effect and favored regeneration of the cognate cone type following UV cone ablation. We observed similar results with our UV Kaloop line using methods described above to characterize the blue cone ablation. Approximately 80% of UV cones expressed the nfsb-mCherry transgene (Fig 3). We observed no toxic bystander effect when we performed a TUNEL cell death assay (Fig 6) or photoreceptor counts (Fig 7), similar to the conditional blue cone ablation transgenic system described immediately above.

Overall this characterization argues that both of our newly introduced blue and UV kaloop models of conditional cone ablation are able to ablate a majority of the respective cell class. Further, the ablation appears to primarily be cell autonomous because we could detect no evidence of effects on the nearby neighbour cone cells of other classes.

### Loss of visually mediated behaviour as measured by OMR

**Fig 8:**
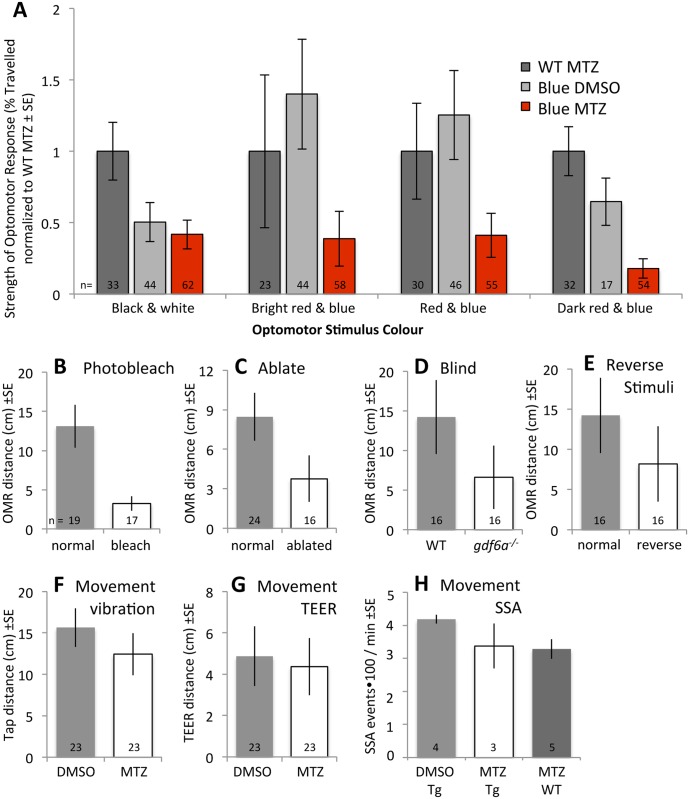
Validation of OMR stimulus as a visually mediated behaviour. The response of zebrafish larvae to our OMR stimuli comprised of blue and dark red bars was used as a measure of visual function. We validated this conclusion in several ways. **A.** 24 hours following drug application on transgenic fish (= blue cone ablation, red bars), responses of zebrafish larvae were observed relative to control fish (grey bars) with various stimuli. Stimuli are detailed in S1 Fig, and consisted of typical OMR stimuli of black & white bars, or alternatively blue bars with intervening red bars of various saturation. All stimuli presented were able to distinguish the visually mediated behaviour of larval zebrafish with blue cones ablated relative to controls. The blue and dark red stimuli pair evoked responses that were least variable between individuals and showed the largest magnitude of change to both control treatments, and thus was selected as the stimulus for the majority of our work. **B.** Larvae acutely blinded by UV light, presumably bleaching their photopigments, responded less than their unblinded siblings (p<0.05). **C.** Larvae showed photoreceptor degeneration one day following blinding by intense UV light (S2 Fig) and responded less than their unblinded siblings (p<0.05). **D.** Blind fish have reduced responses to our OMR stimulus. *gdf6a*^*-/-*^ mutants, which have previously been shown to exhibit reduced OMR response with typical stimuli, presumably due to overt microphthalmia and cone photoreceptor degeneration, responded less to our OMR stimulus relative to their normophthalmic sighted *gdf6a*^*+/-*^ siblings (p<0.05). **E.** Larvae responding to our OMR stimuli moved through the arena more when stimuli were presented in a typical fashion (described in S1 Fig) compared to when stimuli were presented moving in the opposite direction (p<0.05 by t-test). The latter was predicted to stimulate larvae to remain in their initial position, thus reducing the distance between their initial and final positions, and this prediction was met. **F.** Alternative stimuli were applied to test for any potential movement deficits induced by combining our transgene and MTZ prodrug. A tapping stimulus delivered to the same computer monitor (that was otherwise used to deliver OMR stimuli) induced equivalent amounts of movement in *Tg[sws2*:*nfsb-mCherry]* larvae treated with MTZ (= “Blue MTZ” in later figures) as in their siblings treated with DMSO (p = 0.32). **G.** As per panel F, but potential movement deficits were assessed by Touch Evoked Escape Response (TEER). Transgenic larvae with blue cones ablated did not move less than larvae treated with vehicle DMSO (p = 0.34). **H.** Spontaneous Swim Assay records movement of 10 fish per trial for ten minutes (see S3 Fig). Transgenic *Tg[sws2*:*nfsb-mCherry]* larvae treated with MTZ did not move less compared to when treated with DMSO or compared to wild type treated with MTZ. Statistical comparisons were made via t-test. Sample sizes (n = number of larvae individually tested, except in panel H sample sizes = number of groups of ten larvae tested) are indicated.

To investigate the functional recovery of vision following cone photoreceptor death, ablation was induced in larval zebrafish at 7dpf by exposure to MTZ for 24 hours. After treatment, larval zebrafish underwent behavioural analysis to screen for defects in visual ability (as a result of MTZ-induced cone ablation) that could also be used to assess functional recovery. The OMR utilized a moving bar pattern to assess the visually mediated behavioral response of transgenic zebrafish. Various colors and intensities were utilized to design a stimulus pattern for the behavioural assay (Figs 8A and S1). By adjusting the spectral output of the moving bars, we empirically determined a stimulus that could generate a robust differential behavioral response, as measured by OMR, before and after prodrug treatment. In our hands, the stimulus that yielded the strongest difference between treated and non-treated transgenic fish was a dark red and blue bar pattern (Fig 8A; see also S1 Fig). This stimulus provoked a robust response in non-treated transgenic fish, and failed to do so after treatment. As this color scheme may seem unintuitively effective, we emphasize that the goal during optimization was not to find a stimulus that provoked a maximal effect in *intact* larvae; rather, we aimed to discriminate between presence and absence of blue cones. Although our empirical assessment of such stimuli was never intended to be exhaustive (and was constrained by the potential spectral output a 3 pixel based computer monitor), it is of interest to note that the stimulus we found most effective has low contrast relative to the black and white stripes of traditional OMR or OKR paradigms, and instead displays bars that differ in their colour.

**Fig 9:**
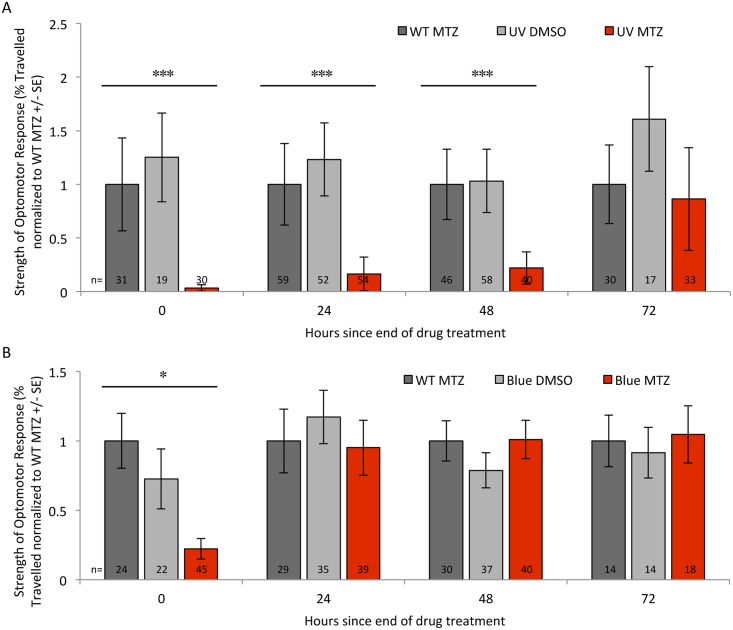
Visually mediated behaviour is reduced following ablation of UV or blue cones but recovers rapidly following blue cone ablation. Thus the recovery of visually mediated behaviour has a time course that depends on the type of cone photoreceptor ablated. Ablation of UV or Blue cones leads to the immediate impairment of visual function (panels A & B, respectively; the first triplet of histograms in each shows behaviour severely decreased following cone ablation (red bars) compared to controls (grey bars)). Visually mediated behaviour regenerated following UV cone ablation over the course of several days (A). Unexpectedly, vision is rapidly restored following ablation of blue cones (B; e.g. red bar at 24 hours since the end of drug treatment). We designed and empirically optimized a visually evoked behaviour based on the optomotor response (OMR; Figs 8A and S1) that stimulates a quantifiable directional movement of freely swimming larval zebrafish. “WT MTZ” are wild type zebrafish receiving the prodrug metronidazole (MTZ) as a control treatment, presented in dark grey bars; “UV DMSO” or “Blue DMSO” in light grey bars are a second control, representing transgenic fish that express nitroreductase for ablation of UV or blue cones, respectively, and these fish received vehicle control (DMSO) only; “UV MTZ” or “Blue MTZ” in red bars are transgenic fish treated with prodrug MTZ and thus had their UV or Blue cones ablated, respectively, immediately prior to testing of visual ability at time zero. Sample sizes (n = number of larvae tested) are reported at the bottom of each bar, and are detailed in Table 2. Table 2 also reports the data prior to the normalization used to generate this Figure. ***p<0.001 experimental relative to controls, *p<0.05 experimental compared to the wild-type control. The visually mediated OMR behaviour had significantly recovered to be indistinguishable from controls 72 hours after UV cone ablation **(A)**, and 24 hours following blue cone ablation **(B)**.

Transgenic larval zebrafish had a significantly reduced response to this stimulus immediately following ablation of UV cones relative to controls (decreased to about 5% of control levels, p<0.001, Fig 9A). These deficits were limited to fish with cones ablated, because transgenic fish receiving prodrug had substantially lower responses compared to either wild type fish receiving prodrug MTZ or compared to transgenic fish receiving DMSO vehicle only (Fig 9A, triplet of histograms at time zero, p<0.05; see also Table 2 for data prior to normalization that was used to generate Fig 9). Ablation of blue cones showed similar deficits in visually mediated behavioural response immediately after ablation (Table 2 and Fig 9B, triplet of histograms at time zero, p<0.05). This supported, for the first time, that the zebrafish visual system is impaired following the specific ablation of either UV or blue cones, and also established a tractable paradigm to subsequently assess functional recovery of vision.

**Table 2:** Visually mediated behaviour is lost following ablation of UV or Blue cones (top & bottom half of Table, respectively) but recovers rapidly following blue cone ablation. This data, after normalization, is also plotted in Fig 9.

**UV cone ablation****:**				
	**Hours since end of drug treatment**			
	**0**	**24**	**48**	**72**
**WT in MTZ** ^1^	40 ± 17 (31)	47 ± 18 (59)	52 ± 17 (46)	35 ± 13 (30)
**Tg(UV) in DMSO** ^2^	50 ± 16 (19)	59 ± 16 (52)	54 ± 15 (58)	56 ± 17 (17)
**Tg(UV) in MTZ**	** 1 ± 1 (30)	** 8 ± 7 (54)	** 12 ± 8 (40)	30 ± 16 (33)
**Blue cone ablation****:**				
	**Hours since end of drug treatment**			
	**0**	**24**	**48**	**72**
**WT in MTZ**	38 ± 7 (24)	33 ± 7 (29)	51 ± 7 (30)	52 ± 11 (14)
**Tg(Blue) in DMSO**	28 ± 8 (22)	39 ± 7 (35)	40 ± 6 (37)	48 ± 10 (14)
**Tg(Blue) in MTZ** ^3^	* 8 ± 3 (45)	32 ± 7 (39)	51 ± 7 (40)	57 ± 11 (18)

^1^ Wild type (WT) fish treated with ablation prodrug metronidazole (MTZ). Data presented as means of fish movement (presented as % of total possible movement) tracking visual stimuli (red & blue moving bars) ± standard error. Sample size (= number of fish) is presented in parentheses.

^2^ Transgenic fish expressing nitroreductase in UV cones [Tg(UV)] treated with vehicle control (DMSO).

^3^ Transgenic fish expressing nitroreductase in Blue cones [Tg(Blue)] treated with ablation prodrug metronidazole (MTZ).

* significantly different (p<0.05) from wild type fish in prodrug (WT in MTZ) at the same timepoint as determined by One-way ANOVA and post-hoc Tukey test.

** significantly different (p<0.001) from wild type fish in prodrug (WT in MTZ) at the same timepoint as determined by One-way ANOVA and post-hoc Tukey test.

### Validation of the OMR approach

We validated this OMR paradigm using three strategies, assessing the following three predictions that emanate from the assertion that quantifying the behavioural response to our OMR stimulus faithfully represents the visual ability of the larva: 1) presentation of alternative stimuli, such as those without moving bars stimulating larvae to swim along the length of the arena, will not elicit movement of the larva to the same extent as our OMR stimulus; 2) Our transgenic fish do not have movement deficits when treated with prodrug MTZ; 3) larvae with other visual system deficits, beyond our cone ablation paradigm, ought to exhibit reduced movement when presented with our stimuli. A fourth type of validation, discussed later, was performed by assessing if alternate assays of photoreception (body pigment dispersion, considered later in these Results) report deficits and recovery coincident to those found in the OMR assay.

First, validating the OMR assay by applying alternative stimuli compared the response of larvae when the red and dark blue bars were played in the opposite direction of our standard OMR (described in S1 Fig), which ought to reduce their total movement if the OMR is valid. Indeed OMR stimuli played in reverse significantly reduced total movement (Fig 8E). Separately, the sum total of *Tg[sws1*:*nfsb-mCherry]* larvae individually tested in variants of red and blue bars was substantial (n = 167 receiving MTZ, n = 192 control larvae, Fig 8A) and their mean response was consistently reduced relative to control fish.

Second, we considered an alternate explanation for our data: that our treatments reduced the overall movement/locomotion of our larvae. We focused our efforts on the blue cone ablation fish, as the transient decrease and rapid recovery of OMR response in these treatments (described below) was not expected. Movement deficits were not suggested by the qualitative observation of the larval behaviour. Neither MTZ treatment itself nor transgenesis itself were sufficient to reduce movement, because control experiments considering these individual treatments were thoroughly shown to have normal responses (Table 2, S1 Table, all grey bars in Figs 8A and 9). Regardless, experiments were performed attempting to directly prove the existence of a movement deficit in our transgenic larvae receiving prodrug. OMR using black and white stimuli performed on larvae lacking blue cones failed to show a difference in movement compared to controls (S1 Table). Next, *Tg[sws2*:*nfsb-mCherry]* larvae were tested in the identical troughs to the OMR paradigm but were presented with a blank screen and given a tapping (acoustic/vibrational) stimulus instead. These larvae moved through the trough towards the tapping stimulus, and larvae with and without DMSO did not show significant differences in movement (Fig 8F; n = 23). Similarly treated larvae were individually placed in nearly identical conditions, but held instead in a petri dish to measure the touch evoked escape response. *Tg[sws2*:*nfsb-mCherry]* larvae with and without DMSO did not show significant differences in movement (Fig 8G; n = 23). A third movement assay again compared *Tg[sws2*:*nfsb-mCherry]* larvae with DMSO vs. MTZ in a similarly positioned petri dish, but assayed spontaneous movement of a group of 10 larvae at a time, very similar to the Spontaneous Swimming Assay described previously [45]. Again no significant deficits in movement were observed (Fig 8H; n = 3–5 with 10 larvae in each sample). Thus larvae maintained in conditions very similar to our OMR assay, but receiving numerous alternative sensory stimuli to induce movement (touch, acoustic, vibration, multimodal interactions with conspecifics) all failed to reveal any significant movement deficit when blue cones were ablated.

**Fig 10:**
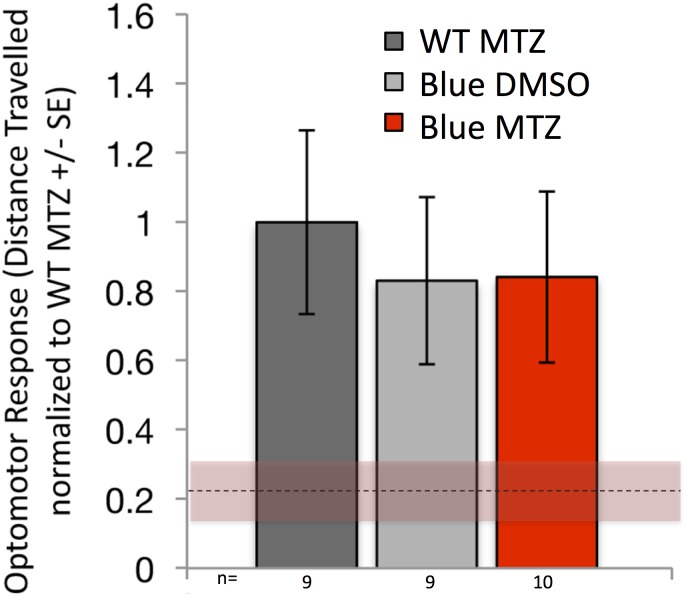
Rapid functional recovery of visually evoked behavioural response is not dependent on blue cone generation or regeneration. Blue cones were ablated in larvae, which were treated with the prodrug metronidazole (MTZ) for 48 hours beginning at 7 dpf, then behaviourally tested immediately following the removal of the prodrug. This treatment was intended to kill any blue cones that were potentially being generated during the 24–48 hours-post-ablation window, which was when behavioural recovery unexpectedly occurred in Fig 9. Dotted line & shading represent the mean ± SE of OMR response at 24 hours post ablation, i.e. is a benchmark of reduced visually mediated behaviour immediately following cone ablation derived from the red shaded bottom left histograms in Fig 9B. Despite this extended treatment of prodrug MTZ that prevented any putative regeneration/addition of blue cones, visually-mediated behaviour fully and rapidly recovered (compare red bar to grey controls, no significant difference) Figure legend as per Fig 9, such that red bars represent fish with blue cones ablated and grey bars represent control treatments. “WT MTZ” are wild type zebrafish receiving the prodrug metronidazole (MTZ) as a control treatment, presented in dark grey bars; “Blue DMSO” in light grey bars are a second control, representing transgenic fish that express nitroreductase for ablation of blue cones that received vehicle control (DMSO) only; “Blue MTZ” in red bars are transgenic fish treated with prodrug MTZ and thus had their Blue cones ablated. n = 9–10 fish per bar for the 48 hour treatment, as detailed in S2 Table that also reports the data prior to the normalization used to generate this Figure. The data are from fish comparable to those in the second set of histograms in Fig 9B, wherein visually-mediated behaviour had returned to control levels 24 hours after cone ablation; here, MTZ was kept on the larvae for an additional 24h (48h total) so that the rapid recovery of vision could be assessed while the addition/regeneration of blue cones was blocked. The recovery of vision to control levels in these conditions demonstrates that addition of blue cones (e.g. via regeneration or proliferation at the CMZ) is not required for the rapid recovery of vision reported in Fig 9B.

A final test of movement was derived from experiments modulating the duration of MTZ exposure: If MTZ were to have a negative impact on the movement of our transgenic larvae then extending the duration of MTZ ought to reveal either an extended deficit in OMR response or a greater magnitude of effect (even lower) OMR response. What we observed instead, starkly contrasting these predicted outcomes, is that *Tg[sws2*:*nfsb-mCherry]* larvae receiving 48 hours of MTZ treatment had fully recovered their OMR response, contrasting those exposed to 24 hours of MTZ that showed reduced OMR (Fig 10). Thus multiple lines of evidence fail to support the contention that our transgenic larvae treated with MTZ exhibit decreased overall movement, except of course when said movement is stimulated by visual processes such as in our OMR assay.

Third, we tested whether our OMR assay with blue and dark red stimuli could reveal deficits in larval zebrafish with known vision deficits. We used age-matched microphthalmic *gdf6a*^*-/-*^ larvae because they have a decrease in abundance of blue cones [11] and have been shown to respond poorly to more traditional OMR stimuli [46]. Microphthalmic *gdf6a*^*-/-*^ larvae responded to our OMR stimuli significantly less than their normophthalmic siblings (Fig 8D). A second approach was to blind larvae using a bright UV light applied from the same position (ventrally) as that in the OMR, with the presumption that it would bleach retinal photopigments and render the fish temporarily blind. This treatment, applied immediately prior to the OMR assay, significantly decreased response to the OMR stimulus (Fig 8B). These larvae were subsequently left to recover for a day and retested in OMR, as we expected this bright UV light would ablate retinal photoreceptors. These larvae also exhibited significantly decreased response to the OMR (Fig 8A). Histology revealed that this treatment had indeed ablated photoreceptors, reminiscent of similar treatments on adult [48, 49] fish, including truncated photoreceptor outer segments and the appearance of pyknotic nuclei only in the outer nuclear layer (S2 Fig). Thus multiple types of vision deficit are all detected by our OMR stimulus and methods, consistent with the conclusion that blind fish cannot see.

Finally, we describe below that other metrics of ocular photoreception (changes in body pigmentation upon application of UV light) are reduced and subsequently recover following UV cone ablation on a timeline nearly coincident with the OMR results we observe.

Thus the modulations of OMR responsivity following ablation of UV or Blue cones in our transgenic fish appear to represent bona fide visual deficits and subsequent recovery.

### Functional recovery of visual behaviour following UV cone ablation

We next characterized the recovery of visually mediated behaviour following cone ablation, expecting from past work that regeneration of cones typically occurs over the course of several days [16]. As described above, the visually mediated behaviour of zebrafish was substantially and significantly reduced following UV cone ablation in comparison to control groups (p<0.05) (Table 2 and Fig 9A, time zero, i.e. immediately following ablation). Recovery of visually mediated behaviour occurred gradually: after 72 hours of recovery the UV cone ablated fish had functionally recovered the visually evoked behaviour to be indistinguishable from control levels. Visually mediated behaviour was not assessed beyond this time, as the fish reached an age where they are variably in poor condition if not transferred to rearing facilities designed for juvenile fish. Histological sections taken at 72 hours post-ablation demonstrate that the incremental appearance of healthy UV cones at the margins of the retina correlates with the functional regeneration of visual response (Fig 3).

### Rapid recovery of visual behaviour following blue cone ablation

Considering the gradual recovery of visual behaviour following UV cone ablation described immediately above, we predicted a similar progressive recovery of behavioural response following blue cone ablation. As described above, the visually mediated response was significantly reduced immediately following blue cone ablation (Table 2 and Fig 9B, time zero, p<0.05). However, functional recovery of visually evoked behaviour occurred much more quickly than expected; after a 24-hour recovery period the magnitude of behavioural response in transgenic fish receiving prodrug MTZ was indistinguishable from either control group (Fig 9B), and this recovery was robustly represented in repeated measures on subsequent days. This rapid timeframe for functional recovery was unexpected based upon the time course of cone regeneration from past work, and considering that histological sections show that transgene expressing blue cones were not present so early following cessation of drug treatment (Fig 2).

Loss and recovery of visually mediated behaviour following ablation of UV cones was similar to that described above when the fish were presented with black and white moving bars (S1 Table). Thus the red and blue bars presented as stimuli above, which we had determined empirically to maximally detect loss of vision following short wavelength sensitive cone ablation, are effective, but other high contrast stimuli can also be used to assess some disruptions and recovery of visually mediated behaviour in our paradigm.

### Rapid recovery of visual function is independent of blue cone regeneration

The rapidity of visual function recovery following blue cone ablation led us to speculate that cones might be rapidly replaced into the retina following ablation, either via stem cells supporting cone regeneration, or via addition of new cones at the continuously proliferating ciliary marginal zone.

To test if such regeneration or differentiation of blue cones was responsible for the rapid functional recovery of vision, we extended the prodrug MTZ treatment to obviate any theoretical generation of blue cones. Prodrug treatment exposure was extended to 48 hours, thereby encompassing the recovery window in the previous experiment. Despite this prolonged drug exposure, visual function recovered quickly. Following 48 hours of prodrug treatment, visually mediated behaviour was indistinguishable compared to control fish (different neither to the wild type treated with MTZ nor to the transgenic zebrafish treated with vehicle) (n>9 fish per treatment; Fig 10 and S2 Table). These results demonstrate that, after blue cone ablation, functional recovery of OMR response occurred independent of any blue cone photoreceptor generation, because all such generation would have been negated by the extended prodrug treatment. Therefore regeneration of blue cones is not required for the rapid recovery of visually-mediated behaviour observe in this system.

### Decreased photoreception of UV light following UV cone ablation measured by changes in body pigmentation

To further confirm that ablation of cones affects ocular photoreception, as suggested by the OMR data above, we sought paradigms to measure ocular photoreception in larval zebrafish that are inert to potential confounds related to potential deficits in neuromuscular system and larval movement. We utilized the recent observation that UV light induces a darkening of the body pigmentation via melanin dispersion [50], a response that is presumably adaptive to protect the fish from damaging UV light. We deployed a version of this assay by dark-adapting larvae and quantifying changes to pigmentation upon application of UV (405 nm) light from below the fish, quantifying the melanin dispersion using established methods [51]. The role of ocular photoreceptors in mediating this response to UV light has been affirmed in multiple ways, especially including that the response is truncated: i) when eyes are surgically enucleated; ii) when eyes are absent due to mutations in the gene *rx3*; iii) when UV cones are largely absent from the retina, such as in tbx2b^lor^ mutants; and iv) when tails are isolated from the head [50]. We reasoned that if the loss and recovery of visually mediated behaviour inferred from the OMR results above were valid, it would be correlated with the timecourse of photoreception deficits and recovery on other assays.

**Fig 11:**
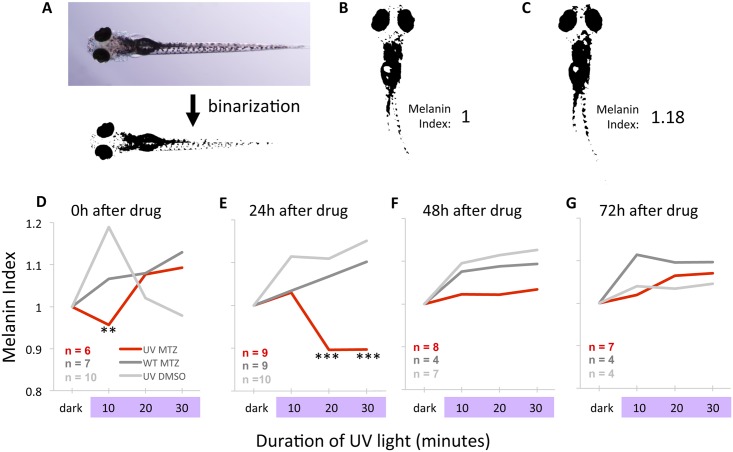
Photoreception of UV light is reduced following ablation of UV cones as measured by changes in body pigmentation. UV cones were ablated in larvae, and larval body pigmentation was measured in response to UV light; zebrafish larvae of this age are known to darken in response to UV light via melanin dispersion. **A.** Dorsal view of larval zebrafish. Binarization of images was used to quantify melanosome reaction to UV light. Larvae were imaged at multiple time points, beginning with dark-adapted larvae. **B.** The number of pixels in dark-adapted larvae was given a melanin index of 1. **C.** UV light exposure causes wild type fish to darken, increasing the melanin index. **D.** Shortly after drug treatment (0 hours), control larvae respond to UV light with the expected darkening of body pigmentation (grey lines), whereas larvae with UV cones ablated exhibit a significantly delayed response (red line). **E.** Larvae tested 24h after drug treatment reveal that UV cone ablation significantly impairs photoreception. **F,G.** Larvae tested 48 and 72 hours after drug treatment reveal a gradual recovery of sensitivity to UV light to levels indistinguishable from controls. ** = p<0.01 compared to UV+DMSO group *** = p<0.001 comparing UV+MTZ group to larvae in all control groups in the same timepoint, and these data are also significantly different (p<0.01) from the melanin index of the same UV+MTZ larvae in the dark-adapted state. Statistical significance was determined by Two-way ANOVA with a post-hoc Tukey test. The standard error surrounding each mean is reported in Table 3.

As expected, melanin dispersion occurred in larval zebrafish exposed to UV light (grey lines, Fig 11). This assay begins with dark-adapted fish wherein melanin is already largely dispersed, so changes in pigmentation are not large, but they are robust (Table 3). Transgenic larval zebrafish had a significantly reduced response to this stimulus immediately following ablation of UV cones relative to both controls (p<0.01), indeed demonstrating a transient decrease in melanin dispersion (= lighter body appearance) in response to UV light rather than the expected increase in melanin dispersion. This differential response of transgenic larval zebrafish relative to controls was of greater magnitude and duration 24 hours after MTZ treatment, such that responses to UV light were significantly lower when UV cones were ablated (p<0.001, Fig 11 and Table 3). Further, the pigment dispersion at this timepoint was significantly less than that of the same transgenic larvae in the dark (p<0.01), which was not observed in any other set of conditions. These deficits were limited to larvae with cones ablated, because transgenic fish receiving prodrug had significantly lower responses compared to either wild type fish receiving prodrug MTZ or compared to transgenic fish receiving DMSO vehicle only.

**Table 3:** Larval zebrafish body pigmentation changes during exposure to UV light and is modulated by UV cone ablation ^1^.

		**Hours since end of drug treatment**							
		**0h**				**24h**			
*UV light (min)*		0	10	20	30	0	10	20	30
Group	UV MTZ	1 ± 0.04 (6)	0.96 ± 0.04 (6) **	1.08 ± 0.05 (6)	1.09 ± 0.09 (6)	1 ± 0.05 (9)	1.03 ± 0.02 (9)	0.90 ± 0.02 (9) ***	0.90 ± 0.02 (9) ***
	UV DMSO	1 ± 0.12 (10)	1.19 ± 0.06 (10)	1.02 ± 0.07 (10)	0.99 ± 0.04 (10)	1 ± 0.12 (10)	1.11 ± 0.04 (10)	1.11 ± 0.05 (10)	1.15 ± 0.05 (10)
	WT MTZ	1 ± 0.10 (7)	1.07 ± 0.02 (7)	1.08 ± 0.08 (7)	1.13 ± 0.06 (7)	1 ± 0.03 (9)	1.03 ± 0.01 (9)	1.07 ± 0.01 (9)	1.10 ± 0.02 (9)
		**Hours since end of drug treatment**							
		**48h**				**72h**			
*UV light (min)*		0	10	20	30	0	10	20	30
Group	UV MTZ	1 ± 0.06 (8)	1.02 ± 0.02 (8)	1.03 ± 0.03 (8)	1.11 ± 0.04 (8)	1 ± 0.08 (7)	1.02 ± 0.01 (7)	1.07 ± 0.02 (7)	1.07 ± 0.02 (7)
	UV DMSO	1 ± 0.09 (7)	1.10 ± 0.03 (7)	1.11 ± 0.04 (7)	1.13 ± 0.02 (7)	1 ± 0.09 (4)	1.04 ± 0.03 (4)	1.03 ± 0.04 (4)	1.05 ± 0.06 (4)
	WT MTZ	1 ± 0.06 (4)	1.08 ± 0.02 (4)	1.09 ± 0.02 (4)	1.09 ± 0.0 (4)	1 ± 0.04 (4)	1.11 ± 0.04 (4)	1.10 ± 0.04 (4)	1.10 ± 0.04 (4)

^1^ Values represent Mean ± 1 SE. Numbers in brackets represent the sample size, i.e. the number of larvae assessed. The means of this data are plotted in Fig 11.

** = p<0.01 compared to UV+DMSO group as determined by two-way ANOVA with a post-hoc Tukey test.

*** = p<0.001 comparing UV+MTZ group to larvae in all control groups in the same timepoint, and these data are also significantly different (p<0.01) from the melanin index of the same UV+MTZ larvae in the dark-adapted state. Determined by Two-way ANOVA with a post-hoc Tukey test.

Following the ablation of UV cones in transgenic fish by application of MTZ, these deficits in ocular photoreception, as measured by pigment dispersion, slowly recovered. At 48 hours the pigment dispersion was observed to be of intermediate magnitude relative to cone ablation and control larvae, and significantly different from neither (Fig 11 and Table 3). By 72h post-ablation the melanin dispersion had recovered to levels indistinguishable from either control group (Fig 11 and Table 3). This timeline of ocular photoreception deficits measured by pigment dispersion is in good concordance with the visual deficits measured by OMR (compare Fig 11D–11G with the groups of histograms in Fig 9A). The coincidence of timing is imperfect, with the largest magnitude of deficits being offset by the apparently slower degeneration of the pigment assay, though the low temporal resolution (one set of measurements per day of recovery from cone ablation) hampers a conclusive comparison in this regard.

In sum, the assessment of ocular photoreception via measuring body pigment dispersion concurs with results from the OMR in assessing vision following UV cone ablation. It supports several conclusions: i) it is a further (fourth) type of validation that our novel OMR stimulus is inducing visually mediated behaviour and faithfully reporting retinal photoreception and vision; ii) the coincidence of timing in photoreception deficits and recovery following UV cone ablation, as revealed by each assay, suggests that retinal photoreception is able to recover to normal levels in the subsequent days.

## Discussion

Principal hurdles in deploying stem cells to repair blindness include, but are not limited to, two challenging issues: First, stem cells must be directed to differentiate into cones because restoration of daytime and high-acuity vision is a highly desirable patient outcome; Second, visual function must be restored, thus the retina and regenerated cones require plasticity to develop, survive and rewire; the mechanisms of such plasticity and how to best affect it remain elusive. Zebrafish offer distinct advantages towards the first issue, and will have increasing potential to contribute to the second issue when assays of visual function in zebrafish mature and are made tractable and accessible.

Here we develop two novel transgenic models of conditional cone photoreceptor ablation and compare the resultant visual function, with special focus on loss and recovery of an experimentally tractable visually mediated behaviour. We chose to ablate UV cones, improving on our recent transgenic models [16] by increasing penetrance and longevity of transgene expression via Kaloop technology. We chose to ablate blue cones as a comparator because these two cone types share several similarities, including both being of single cone morphology, mediating sensitivity to short wavelength light, and apparently sharing a recent evolutionary history in-so-much that they both express short wave sensitive (*sws)* class opsin genes that are each other’s closest paralogs. We expect that by formally comparing the ablation of multiple photoreceptor types, especially in a conditional, tractable and specific manner, we will be able to observe insightful differences during the biological reaction to cone loss. This would especially include regenerative responses and neuronal plasticity that can be measured (and eventually manipulated) at the molecular, cellular, physiological and behavioural levels. Here we characterize our models and report the first such distinction between responses to cone ablation. Thus, when UV cones were ablated visual function regenerated on a gradual time course (over several days) coincident with addition of UV cones at the CMZ. Surprisingly, however, the loss of visually mediated behaviour associated with death of blue cones rapidly recovered to control levels within 24 hours. After validating our OMR assay and eliminating several alternate explanations, the rapid and robust recovery of function leads us to speculate that a surprising capacity for synaptic plasticity might exist in the remaining cells; the mechanisms of this plasticity remains undetermined but comparison between our models has good potential to offer future insights.

Our results demonstrate that cone photoreceptors expressing the transgenic constructs were efficiently ablated using the nitroreductase/metronidazole method. Functionally, this method provides temporal control of the onset of cell death and regeneration through the controlled exposure of the prodrug with no detectable bystander effect on adjacent photoreceptors. The results of our cell death assay and analysis of photoreceptor abundance allow us to conclude that the nitroreductase/metronidazole method of cell ablation is specific, cell autonomous, and does not cause a deleterious, toxic bystander effect. This is consistent with previous investigations into the nitroreductase system for targeted cell ablation in zebrafish retina [16, 17, 31]. Any potential toxic effect on behavioural responses induced by the prodrug MTZ or by the transgenic engineering were excluded by comparing the results to two control treatments: wild type fish receiving prodrug MTZ and transgenic fish of each type receiving vehicle control only. Behavioural responses were similar between these controls and remained constant throughout the duration of the experiment.

### Melanin dispersion is mediated by UV cone photoreceptors

The melanin dispersion assay measures changes to body pigmentation in response to UV light as a measure of ocular photoreception that is not dependent on larval swimming ability. The responses measured by this assay are mediated by photoreceptors in the eye, as established by experiments comparing intact larvae to larvae with eyes removed or isolated body tissue.

Ablation of UV cones led to reduced pigment dispersion, allowing us to conclude that UV cones are required for neural control of body pigmentation. Alternative photoreceptors in the retina playing such a role include rods, other cones, ipRGCs and cryptochromes; outside the retina such photoreceptors could include the pigment cells themselves and the pineal. A similar conclusion to ours was recently attained by measuring melanin dispersion in *lots-of-rods* (“*lor*” = *tbx2b*^*lor*^) mutant larvae which almost entirely lack UV cones [50]. Our data complement and confirm this conclusion, especially removing some assumptions inherent in the previous approach. *Lor* mutants have a reduced complement of UV cones from early in development, but also exhibit phenotypes in other photoreceptors, at least including their namesake increase in rod abundance [11, 52] and defects in the pineal development [53]. Because our methods demonstrate that pigment dispersion was affected following acute ablation specifically of UV cones, our data strengthen and complement the previous conclusion.

### Potential utility to study of retinal regeneration and functional recovery

Alternative paradigms examining retinal repair or regeneration have also innovated to limit the breadth and complexity of the photoreceptor death. While many (genetic) interventions can damage photoreceptors, not all paradigms allow the insult to be transient (reviewed in [54]), such as when using the MTZ-nitroreductase system in the current work. Damage that is acute, such as surgery, delivery of toxic pharmacology or a toxic light dose, rarely offers specificity in regards to the type(s) of photoreceptors ablated. Conditional ablation strategies promising specificity of which photoreceptors are ablated include using precise doses of short wavelength light to bias the damage towards UV cones of adult zebrafish [55], using thyroid hormone to precociously induce the loss of UV cones during the natural ontogeny of trout, using MTZ-nitroreductase system to ablate rods [17] or UV cones [16], and using lasers to ablate localized regions of photoreceptors in rabbit [56, 57]. The latter has revealed a robust and underappreciated plasticity at the level of outer retina in mammals, where photoreceptors adjacent to those killed form new connections within the wound and appear to eventually spread to occupy the vacated space. The recovery of retinal photoreception in the rabbit system has been demonstrated using electrophysiology [56, 57]. The plasticity in such instances occurs over the course of days, not disparate from our observations in zebrafish.

The current work contributes a tractable method for assessing how interventions affect visual recovery, by empirically determining a stimulus that allows us to sensitively determine loss of visual function when either of two short wavelength cones is ablated. The results of our behavioural analysis for UV cone ablation demonstrate that recovery of visual function occurs steadily over 72 hours. Indeed, at 72 hours post UV cone ablation, the behaviour of such fish was not statistically different than wild type control fish. Recovery of visually mediated behaviour coincides with the appearance of UV cones, predominantly observed in the periphery of the retina supporting that the recovery of visually-mediated behaviour is likely due to the generation of UV cone photoreceptors. The dorsal aspect of the proliferating ciliary marginal zone, being at the periphery of the retina, is ideally situated to detect the substrate level stimuli we presented (i.e. stimuli below the swimming fish). Thus, functional recovery reported herein could be the combined result of cone regeneration and cone addition as the retina continues to grow. We have yet to resolve whether compensatory proliferation accelerated the recovery of visually mediated behaviour in the current treatments, however very similar interventions led to the induction of Müller glia proliferation following ablation of UV cones [16] or all rod photoreceptors [17]. Regardless, following ablation of UV cones, zebrafish were able to fully recover visual function, and this offers the unique opportunity to intervene via pharmacology and genetic engineering to appreciate mechanisms of retinal plasticity associated with addition of new cone photoreceptors into an existing retinal network.

In contrast to the expected gradual recovery following UV cone ablation, visual function was restored within 24 hours post blue cone ablation. This unexpectedly rapid recovery was not due to the contribution of newly generated blue cones from precursors, since recovery of visually mediated behaviour was independent of prodrug duration and the same quick recovery was observed when cone addition was prevented by prolonging the prodrug treatment to encompass the recovery period. This suggests that another mechanism is responsible for the rapid recovery of visual function following blue cone ablation.

### Speculation on mechanisms of rapid recovery of vision following blue cone ablation

Briefly considering alternative explanations to those immediately above for the quick recovery of vision following blue cone ablation, we speculate that: (1) ablating blue cones may reveal a latent second mechanism to detect these stimuli (moving red & blue bars); (2) Initial reductions in behavioural responses are due to debris of dying cones (not from the lack of cones) and clearance of such debris enables recovery of visual behaviour; (3) Finally, we hypothesize that blue cone ablation induces synaptic remodelling of remaining cones and interneurons to compensate for the loss of afferent input or connectivity. The first alternative is difficult to reconcile with the observation that visually mediated behaviour was dramatically reduced upon ablation of blue cones; thus if any such imagined alternate system exists it appears to require time to establish itself. This brief delay in restoring vision after blue cone ablation is more consistent with the two latter alternates. The second alternative would need to be rationalized against the differential results from UV cone ablation, thereby requiring that debris from dying blue cones be cleared quickly compared to that of UV cones. Considering there is also no evidence that cellular debris can block vision in this manner, this alternate seems unlikely. With no additional alternate explanations yet imagined, we suggest a mechanism of the rapid visual recovery following blue cone ablation might be synaptic plasticity. We speculate that the remaining photoreceptors begin to form new and abnormal connections in the outer retina, and that this abnormal rewiring underpins the ability of the fish to detect the visual stimuli presented. Ongoing experiments will attempt to discern cellular and molecular characteristics of any such plasticity, using the UV cone ablation as a useful comparison.

The suggestion that the remaining rods and cones mediate the quickly regenerated visual response (or lack thereof) holds some intriguing parallels with the spectral sensitivity of the ablated vs. remaining cone classes. For example, the lack of recovery following UV cone ablation is coincident with the fact that UV cone maximal sensitivity sits at an extreme end of the zebrafish visual spectrum; when UV cones are ablated there is only the overlapping sensitivity of blue cones to compensate (though perhaps β-band absorption from green and red cone opsins ought not be discounted entirely; e.g. see [21]). In contrast, lack of blue cones has the potential to be compensated by both the UV cones and green cones that bracket and overlap blue cone sensitivity in both the short and long wavelengths respectively. Rods also have an appropriate spectral sensitivity to play such a role and could be involved in this response, especially if loss of blue cones affected the state of rod adaptation to prevailing light. It remains to be determined whether the rapid recovery of visually mediated behaviour depends upon the activity of adjacent/remaining photoreceptor types following blue cone death.

Considering that zebrafish are tetrachromats and utilize four different cone types [20, 21], it remains possible that the loss of one cone type is compatible with retaining color opponency. Alternatively, the interneurons could be rewiring to the cones of the targeted subtype that survived due to the incomplete penetrance of the transgenes, and the two cone subtypes examined here may have a differing potential for synaptic plasticity. The loss of afferent input is known to affect and bias synaptogenesis, such that, for example, retinal H3 horizontal cells increasingly synapse with blue cones when UV cones are not present, however H3 horizontal cell synaptogenesis is unchanged when blue cones are missing [25]. Our OMR results following specific ablation of short wavelength sensitive cones support previous evidence that, while red and green cones are the dominant inputs mediating the OMR, the presence of short-wavelength sensitive cones is required for the OMR, and may potentially have an important role contributing inhibitory input [58, 59]. This speculation is broadly paralleled by a potential interpretation of our pigment dispersion results, also suggesting an inhibitory role of UV cones on the output of other photoreceptors: Ablation of UV cones altered the physiological response to light from one driven by UV cones (further pigment dispersion, as observed previously [50]) to one driven by long wavelength cone (reduced pigment dispersion) despite the applied light remaining constant. Future work will examine whether ablating blue cones has a similar impact on how body pigmentation reacts to light, or whether further intriguing contrasts can be drawn from the consequences of ablating UV *versus* blue cone photoreceptors.

### Conclusion

We present two novel transgenic zebrafish models of conditional cone photoreceptor ablation. Cone ablation is accompanied by loss of visually mediated behaviour, which slowly recovers to wild type levels as expected when UV cones are ablated. Thus our UV cone ablation model offers the ability to assess how cone addition to the retina is able to mediate functional recovery of vision. In contrast, our second model reveals that visually mediated behaviour recovers rapidly following blue cone ablation. We speculate this rapid recovery is borne upon synaptic plasticity at the level of the outer retina, though future work will be needed to garner evidence to support this. The novel observation of rapid visual recovery following blue cone ablation provides a unique platform to explore mechanisms of regeneration and plasticity at molecular, cellular, and synaptic levels. The model is strengthened by comparison to a parallel model, the ablation of the other short-wavelength single cone subtype (UV cones) that provides a very different time course of recovery in visual response. Uncovering such mechanisms is expected to contribute to addressing remaining hurdles in therapies to reverse vision loss that deploy stem cell therapy to restore daytime visual ability.

## Supporting Information

S1 Table Visually mediated behaviour is lost following ablation of UV cones (top & bottom half of Table, respectively) but recovers in subsequent days. Stimulus presentation is black and white moving bars (as opposed to red and blue bars in Table 2 and Fig 9).
(DOCX)

S2 Table Rapid functional recovery of visually evoked behavioural response is not dependent on blue cone generation or regeneration. No significant differences were found between these treatments.
(DOCX)

S1 Fig Optomotor assay method and characterization of stimuli. **(A)** Optomotor apparatus consists of a horizontally positioned computer screen with narrow troughs containing one zebrafish larva each (see Methods). The behaviour is captured from overhead on a camera. Larvae are individually positioned in the end of small (1 X 30 cm) troughs. In this schematic, the larva would be positioned at the end of the trough closest to the reader, and moving stripes would stimulate the larva to move along the trough away from the reader (yellow arrow). Failure to move along the trough is used as a metric of visual disability. In some experiments used to validate this OMR method, the stimulus direction was reversed (Fig 8E), such that in this schematic the larva would be in the same position, but the stimulus (stripes) would be moving towards the reader. **(B)** Color patterns of the optomotor stimuli (viewed from top) empirically assessed in Fig 8A, and the respective spectral irradiance of each stimulus generated by the LCD computer monitor (bottom).
(TIF)

S2 Fig Ablation of photoreceptors in larval zebrafish by ventral application of bright UV light. One day following ablation, photoreceptors have truncated outer segments, and pyknotic nuclei are observed on the photoreceptor layer. Cryosections stained with TO-PRO-3 to label nuclei and Bodipy to label lipid-rich material such as outer segments.
(TIF)

S3 Fig Example results from Spontaneous Swim Assay. Three petri dishes (100 mm diameter) are displayed, each of which contained 10 larval zebrafish. The detail within each dish/circle is a compilation of all the positions the larvae occupied over ten minutes. Examining the difference in intensity between movie frames provides “movement events”, and the number of these events per minute is an established measure of larval fish movement. No differences in movement were note amongst the treatments, *Tg[sws2*:*nfsb-mCherry]* larvae treated with prodrug MTZ (MTZ Tg) were not significantly different that these transgenic larvae treated with vehicle DMSO (DMSO Tg) or wild type larvae treated with MTZ (WT MTZ) as plotted in Fig 8H.
(TIF)

S4 Fig Proliferation induced by cone ablation. Proliferation was assessed following ablation of UV cones or Blue cones in the respective transgenic fish by application of the prodrug metronidazole (MTZ) and compared to fish receiving vehicle alone (DMSO). EdU (5-ethynyl-2´-deoxyuridine) was added as a bath treatment (e.g. see Fig 1 for time course) and is incorporated into dividing cells. **(A-C)** EdU+ cells (green) displayed typical abundance in the ciliary marginal zone (CMZ), the outer nuclear layer (ONL) and inner nuclear layer (INL) of DMSO treated fish. An increase in EdU+ cells was apparent in the CMZ of larvae where UV or Blue cones had been ablated. **(D-F)** The abundance of EdU+ cells in these three tissue compartments was quantified in each of the DMSO, Blue-MTZ and UV-MTZ treatment groups (n = 9, 6 or 8 larvae respectively) and normalized against the length of the outer plexiform layer. * = p<0.05, ** = p<0.01, *** = p<0.001 by Kruskal-Wallis test.
(TIFF)
