# Supplementary material for: Rapid Recovery of Visual Function Associated with Blue Cone Ablation in Zebrafish
Source: PLoS One. 2016 Nov 28;11(11):e0166932. doi: 10.1371/journal.pone.0166932 (PMC5125653; doi:10.1371/journal.pone.0166932)
Supplement: S1 Table — Stimulus presentation is black and white moving bars (as opposed to red and blue bars in Table 2 and Fig 9). (DOCX) [file pone.0166932.s001.docx]

**S1 Table. Visually mediated behaviour is lost following ablation of UV cones** (top & bottom half of Table, respectively) but recovers in subsequent days. Stimulus presentation is black and white moving bars (as opposed to red and blue bars in Table 2 and Figure 9).

| **UV cone ablation:** | | | | |
| --- | --- | --- | --- | --- |
|  | **Hours since end of drug treatment** | | | |
|  | **0** | **24** | **48** | **72** |
|  |  |  |  |  |
| **WT in MTZ** ^1^ | 47 ± 18 (31) | 50 ± 18 (59) | 52 ± 17 (45) | 33 ± 13 (29) |
|  |  |  |  |  |
| **Tg(UV) in DMSO** ^2^ | 55 ± 15 (31) | 60 ± 16 (65) | 57 ± 16 (60) | 44 ± 16 (17) |
|  |  |  |  |  |
| **Tg(UV) in MTZ** | ** 5 ± 6 (29) | ** 12 ± 7 (60) | * 23 ± 14 (44) | 37 ± 18 (32) |
|  |  |  |  |  |
| **Blue cone ablation:** | | | | |
|  | **Hours since end of drug treatment** | | | |
|  | **0** | **24** | **48** | **72** |
|  |  |  |  |  |
| **WT in MTZ** | 42 ± 8 (26) | 26 ± 5 (50) | 46 ± 7 (40) | 63 ± 8 (24) |
|  |  |  |  |  |
| **Tg(Blue) in DMSO** | 18 ± 5 (39) | 36 ± 5 (47) | 42 ± 5 (48) | 72 ± 7 (25) |
|  |  |  |  |  |
| **Tg(Blue) in MTZ** ^3^ | 16 ± 4 (53) | 25 ± 4 (50) | 32 ± 6 (32) | 62 ± 9 (23) |

^1^ Wild type (WT) fish treated with ablation prodrug metronidazole (MTZ). Data presented as means of fish movement (presented as % of total possible movement) tracking visual stimuli (black & white moving bars) ± standard error. Sample size (=number of fish) is presented in parentheses.

^2^ Transgenic fish expressing nitroreductase in UV cones [Tg(UV)] treated with vehicle control (DMSO).

^3^ Transgenic fish expressing nitroreductase in Blue cones [Tg(Blue)] treated with ablation prodrug metronidazole (MTZ).

* significantly different (*p<0.05; **p<0.001) from wild type fish in prodrug (WT in MTZ) at the same timepoint as determined by One-way ANOVA and post-hoc Tukey test.
