# Supplementary material for: Rapid Recovery of Visual Function Associated with Blue Cone Ablation in Zebrafish
Source: PLoS One. 2016 Nov 28;11(11):e0166932. doi: 10.1371/journal.pone.0166932 (PMC5125653; doi:10.1371/journal.pone.0166932)
Supplement: S2 Table — No significant differences were found between these treatments. (DOCX) [file pone.0166932.s002.docx]

**S2 Table. Rapid functional recovery of visually evoked behavioural response is not dependent on blue cone generation or regeneration.** No significant differences were found between these treatments.

| **Red & Blue bar stimuli:** ^1^ | | |
| --- | --- | --- |
|  |  |  |
| **WT in MTZ** ^2^ | 54 ± 12 (9) |  |
|  |  |  |
| **Tg(Blue) in DMSO** ^3^ | 44 ± 13 (10) |  |
|  |  |  |
| **Tg(Blue) in MTZ** | 42 ± 13 (10) |  |
|  | | |
| **Black & White bar stimuli:** ^4^ | | |
|  |  |  |
| **WT in MTZ** | 51 ± 11 (10) |  |
|  |  |  |
| **Tg(Blue) in DMSO** | 49 ± 13 (10) |  |
|  |  |  |
| **Tg(Blue) in MTZ** | 49 ± 14 (10) |  |

^1^ This data is best compared to the second column of data in Table 2 (bottom half); here fish received 48 hours of drug treatment, whereas in Table 2 the fish received 24 hours of drug treatment. This data, after normalization, is plotted in Figure 10.

^2^ Wild type (WT) fish treated with ablation prodrug metronidazole (MTZ). Data presented as means of fish movement (presented as % of total possible movement) tracking visual stimuli (red & blue moving bars) ± standard error. Sample size (=number of fish) is presented in parentheses.

^3^ Transgenic fish expressing nitroreductase in Blue cones [Tg(Blue)] treated with ablation prodrug metronidazole (MTZ).

^4^ This data is best compared to the second column of data in Supplementary Table S1 (bottom half); here fish received 48 hours of drug treatment, whereas in Supplementary Table S1 the fish received 24 hours of drug treatment.
