# Supplementary figures and images for: Rapid Recovery of Visual Function Associated with Blue Cone Ablation in Zebrafish
Source: PLoS One. 2016 Nov 28;11(11):e0166932. doi: 10.1371/journal.pone.0166932 (PMC5125653; doi:10.1371/journal.pone.0166932)

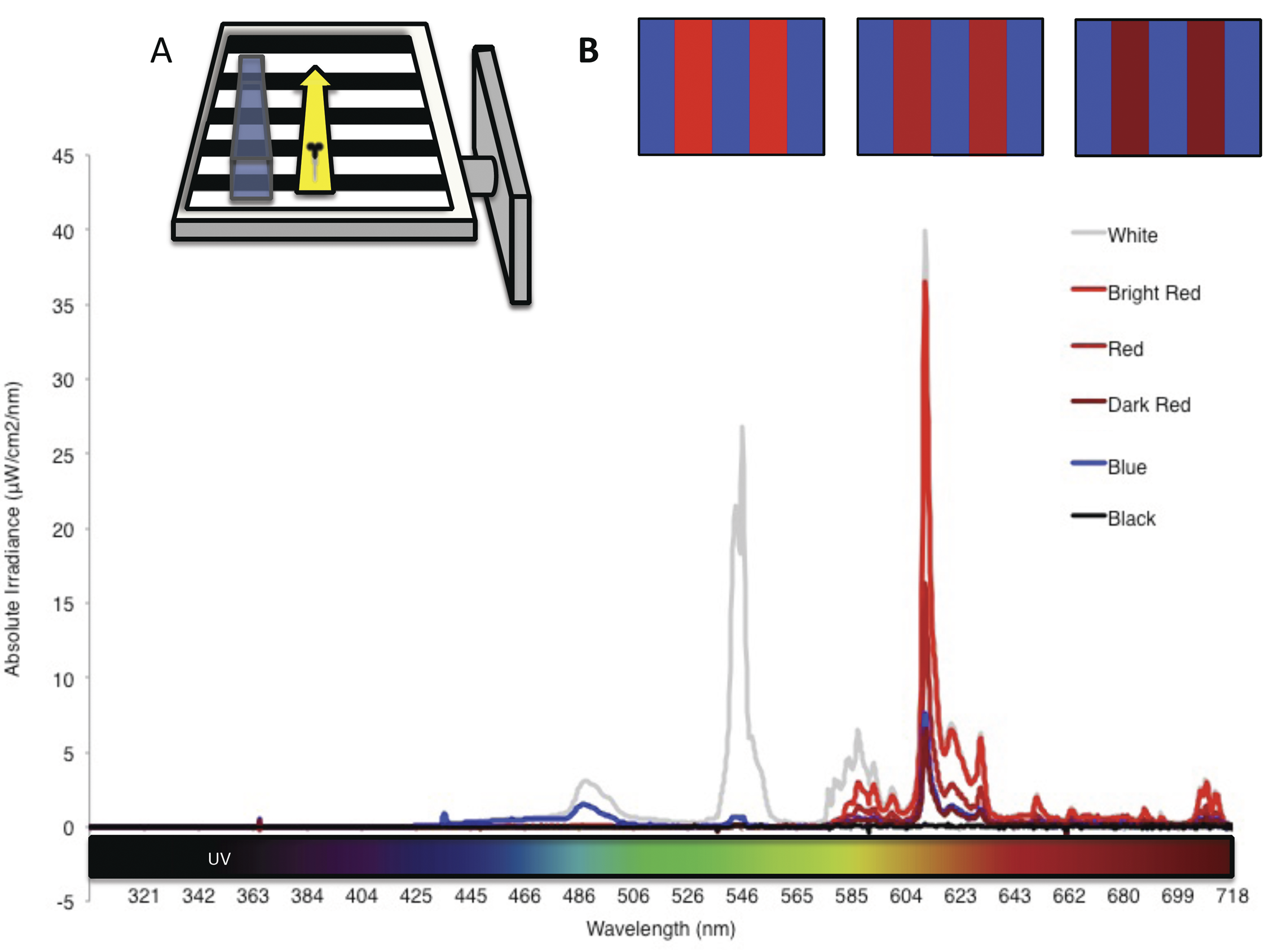

Supplement: S1 Fig — (A) Optomotor apparatus consists of a horizontally positioned computer screen with narrow troughs containing one zebrafish larva each (see Methods). The behaviour is captured from overhead on a camera. Larvae are individually positioned in the end of small (1 X 30 cm) troughs. In this schematic, the larva would be positioned at the end of the trough closest to the reader, and moving stripes would stimulate the larva to move along the trough away from the reader (yellow arrow). Failure to move along the trough is used as a metric of visual disability. In some experiments used to validate this OMR method, the stimulus direction was reversed (Fig 8E), such that in this schematic the larva would be in the same position, but the stimulus (stripes) would be moving towards the reader. (B) Color patterns of the optomotor stimuli (viewed from top) empirically assessed in Fig 8A, and the respective spectral irradiance of each stimulus generated by the LCD computer monitor (bottom). (TIF) [file pone.0166932.s003.tif]

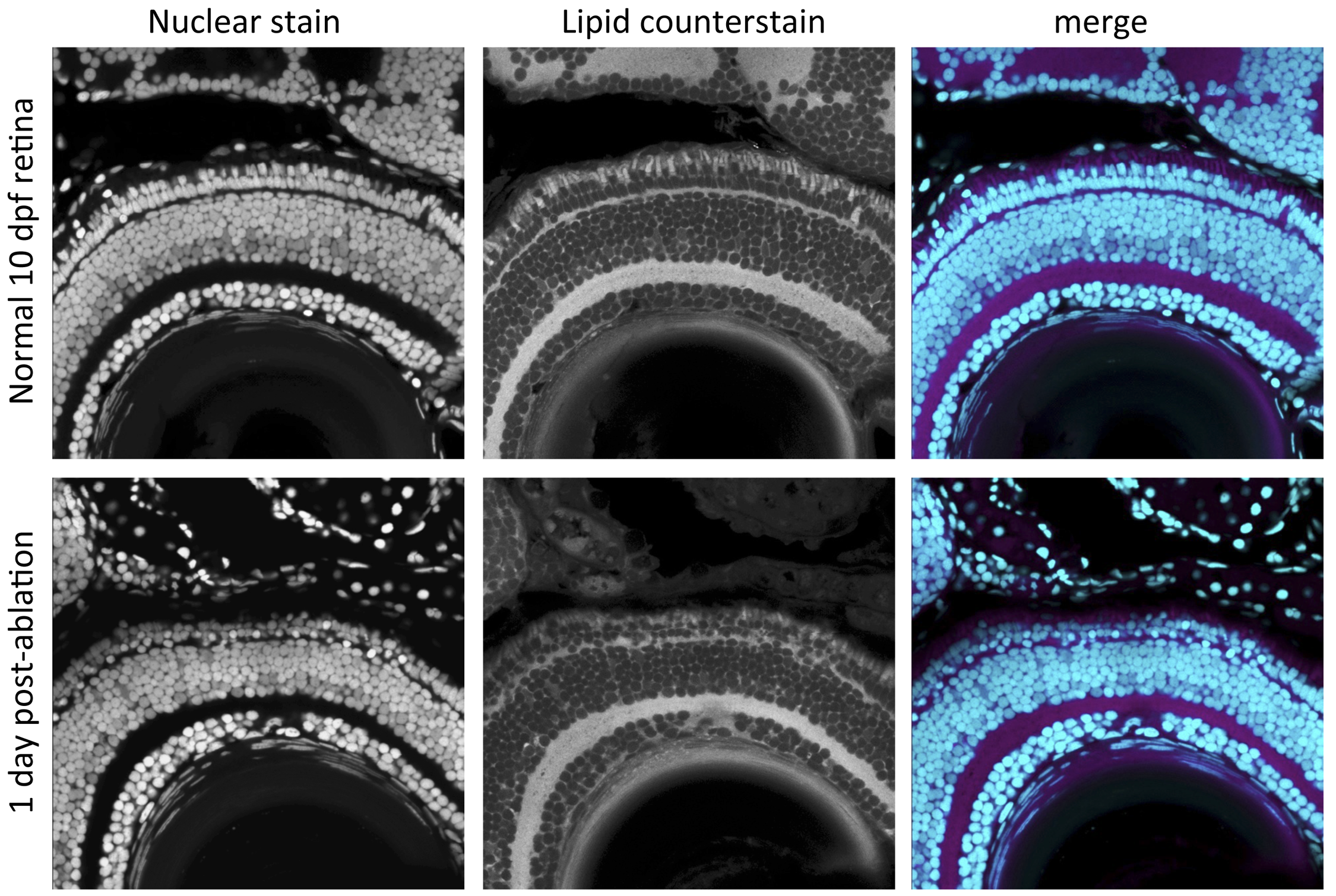

Supplement: S2 Fig — One day following ablation, photoreceptors have truncated outer segments, and pyknotic nuclei are observed on the photoreceptor layer. Cryosections stained with TO-PRO-3 to label nuclei and Bodipy to label lipid-rich material such as outer segments. (TIF) [file pone.0166932.s004.tif]

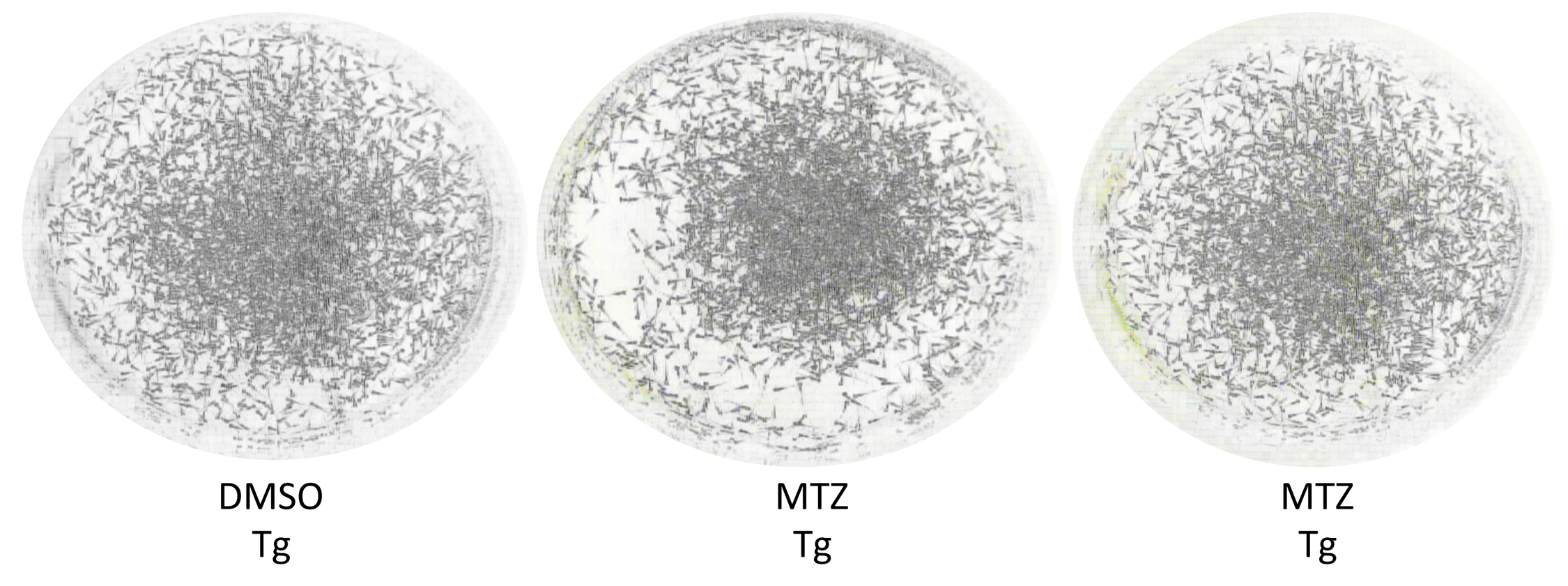

Supplement: S3 Fig — Three petri dishes (100 mm diameter) are displayed, each of which contained 10 larval zebrafish. The detail within each dish/circle is a compilation of all the positions the larvae occupied over ten minutes. Examining the difference in intensity between movie frames provides “movement events”, and the number of these events per minute is an established measure of larval fish movement. No differences in movement were note amongst the treatments, Tg[sws2:nfsb-mCherry] larvae treated with prodrug MTZ (MTZ Tg) were not significantly different that these transgenic larvae treated with vehicle DMSO (DMSO Tg) or wild type larvae treated with MTZ (WT MTZ) as plotted in Fig 8H. (TIF) [file pone.0166932.s005.tif]

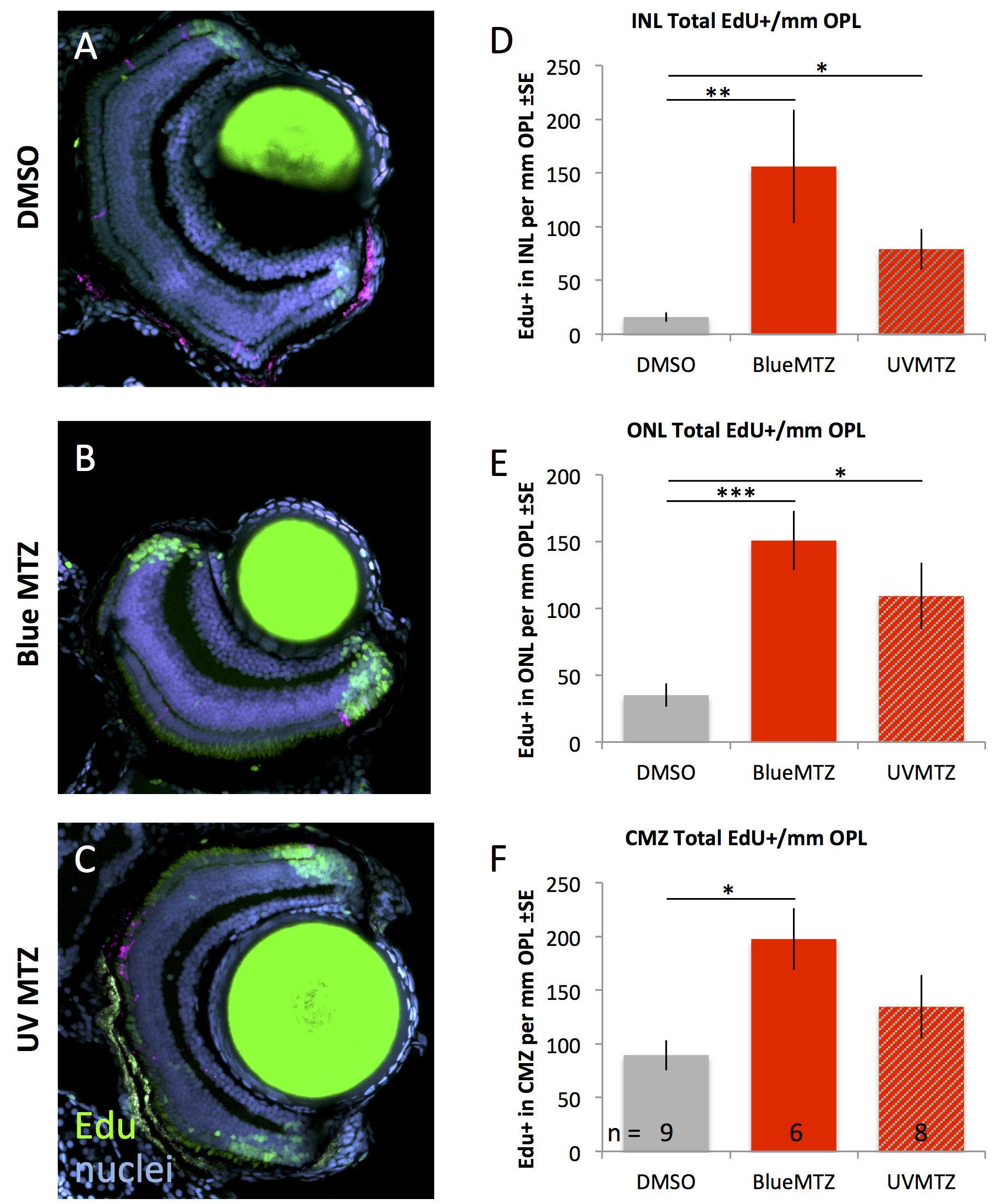

Supplement: S4 Fig — Proliferation was assessed following ablation of UV cones or Blue cones in the respective transgenic fish by application of the prodrug metronidazole (MTZ) and compared to fish receiving vehicle alone (DMSO). EdU (5-ethynyl-2´-deoxyuridine) was added as a bath treatment (e.g. see Fig 1 for time course) and is incorporated into dividing cells. (A-C) EdU+ cells (green) displayed typical abundance in the ciliary marginal zone (CMZ), the outer nuclear layer (ONL) and inner nuclear layer (INL) of DMSO treated fish. An increase in EdU+ cells was apparent in the CMZ of larvae where UV or Blue cones had been ablated. (D-F) The abundance of EdU+ cells in these three tissue compartments was quantified in each of the DMSO, Blue-MTZ and UV-MTZ treatment groups (n = 9, 6 or 8 larvae respectively) and normalized against the length of the outer plexiform layer. * = p<0.05, ** = p<0.01, *** = p<0.001 by Kruskal-Wallis test. (TIFF) [file pone.0166932.s006.tiff]
